# Clinical and genetic heterogeneity of syndromic hearing loss and its non-syndromic hearing loss mimics

**DOI:** 10.1186/s10020-026-01431-6

**Published:** 2026-04-07

**Authors:** Asuman Koparir, Paulina Bahena Carbajal, Mina Zamini, Maryam Naghinejad, Paria Najarzadeh Torbati, Michaela A. H. Hofrichter, Stefanie Tovornik, Erkan Koparir, Neda Dragicevic Babic, Aboulfazl Rad, Daniel Owrang, Irem Kalay, Niloofar Chamanrou, Luis Nicolás Martínez Völter, Nele Christophersen, Tayebeh Baranzehi, Mohsen Rajati, Stephen Loum, Erdmute Kunstmann, Madiha Shadab, Ansar Ahmed Abbasi, Mohammad Doosti, Neda Alidadiani, Shahrooz Ghaderi, Tobias B. Haack, Shahryar Alavi, Julia Doll, Hannie Kremer, Dor Mohammad Kordi-Tamandani, David Murphy, Rahema Mohammad, Helge Hebestreit, Ehsan Ghayoor Karimiani, Sophie Flandin, Paola Linares, Daniel Villalobos, Henry Houlden, Hamid Galehdari, Wafaa Shehata-Dieler, Reza Maroofian, Thomas Haaf, Barbara Vona

**Affiliations:** 1 Institute of Human Genetics, Julius-Maximilians-University Würzburg, Würzburg, Germany; 2 Institute of Medical Genetics, University Hospital Rostock, Rostock, Germany; 3 Department of Biology, Faculty of Science, Shahid Chamran University of Ahvaz, Ahvaz, Iran; 4 Narges Medical Genetics and Prenatal Diagnosis Laboratory, Kianpars, Ahvaz, Iran; 5 Department of Medical Genetics, Faculty of Medicine, Tabriz University of Medical Sciences, Tabriz, Iran; 6 Department of Medical Genetics, Next Generation Genetic Polyclinic, Mashhad, Iran; 7 Department of Biology, Mashhad Branch, Islamic Azad University, Mashhad, Iran; 8 Institute of Clinical Genetics and Genomic Medicine, University Hospital Würzburg, Würzburg, Germany; 9 Center for Rare Diseases, University Hospital Würzburg, Würzburg, Germany; 10 Cellular and Molecular Research Center, Sabzevar University of Medical Sciences, Sabzevar, Iran; 11 Institute for Auditory Neuroscience and Inner Ear Lab, University Medical Center Göttingen, Göttingen, Germany; 12 Auditory Neuroscience and Optogenetics Laboratory, German Primate Center, Göttingen, Germany; 13 Institute of Human Genetics, University Medical Center Göttingen, Göttingen, Germany; 14 Department of Medical Genetics, Umraniye Training and Research Hospital, University of Health Sciences Turkey, Istanbul, Turkey; 15 Department of Plant Production and Genetic Engineering, Faculty of Agriculture, University of Saravan, Saravan, I. R. of Iran; 16 Department of Otolaryngology, Head and Neck Surgery, Tübingen Hearing Research Centre, Eberhard Karls University, Tübingen, Germany; 17 Department of Biology, University of Sistan and Baluchestan, Zahedan, Iran; 18 Department of Zoology, Mirpur University of Science and Technology, Mirpur, Pakistan; 19 Department of Zoology, University of Azad Jammu and Kashmir Muzaffarabad, Muzaffarabad, 13100 Pakistan; 20 Atrak Biotech, Research & Development Division, Bojnurd, Iran; 21 Institute for Medical Genetics and Applied Genomics, University Hospital Tübingen, Tübingen, Germany; 22 Palindrome, Isfahan, Iran; 23 Institute of Pathology, University of Würzburg, Würzburg, Germany; 24 Department of Otorhinolaryngology, Radboud University Medical Center, Nijmegen, The Netherlands; 25 Department of Human Genetics, Radboud University Medical Center, Nijmegen, The Netherlands; 26 Department of Clinical and Movement Neurosciences, UCL Queen Square Institute of Neurology, London, WC1N 3BG UK; 27 Centre for Neuromuscular Diseases, UCL Queen Square Institute of Neurology, London, WC1N 3BG UK; 28 Department of Otorhinolaryngology, Comprehensive Hearing Center, University Clinics, Würzburg, Germany; 29 Universidad Nacional Autónoma de México, Mexico City, Mexico; 30 Department of Bioinformatics, University of Würzburg, Würzburg, Germany; 31 Department of Obstetrics and Gynecology, Brigham and Women’s Hospital, Harvard Medical School, Boston, MA USA; 32 Program in Medical and Population Genetics, Broad Institute of MIT and Harvard, Cambridge, MA USA; 33 Collaborative Research Center 1690 (CRC1690), University of Göttingen, Göttingen, Germany

**Keywords:** Comprehensive genomic testing, Neurodevelopmental disorders, Non-syndromic hearing loss, Non-syndromic hearing loss mimics, Syndromic hearing loss

## Abstract

**Background:**

Hearing loss (HL) is one of the most common congenital conditions and exhibits substantial clinical and genetic heterogeneity. More than 150 genes are associated with non-syndromic hearing loss (NSHL), while over 600 genes are linked to syndromic hearing loss (SHL). Importantly, the absence of additional clinical symptoms at the time of diagnosis does not necessarily exclude SHL. An increasing number of functionally disruptive variants in a growing number of genes have been shown to initially present as isolated HL, only later revealing syndromic features.

**Methods:**

We analyzed clinical data from 111 patients across 102 unrelated families, selected from over 600 individuals negative for *GJB2* and *STRC* variants. Molecular inversion probe panel, exome, or genome sequencing was performed, and patients were retrospectively divided into three subgroups following variant interpretation. Molecular docking was performed on select non-synonymous substitutions.

**Results:**

Subgroup 1 included 30 patients with variants in neurodevelopmental disorder (NDD)-associated genes. HL was the first clinical manifestation in 80% of patients, with it being the sole first symptom in half. Subgroup 2 was comprised of 52 patients with variants in SHL-associated genes unrelated to NDD, while subgroup 3 included 29 patients with variants in genes associated with both NSHL and SHL, such as *SLC26A4* and *USH1C*. In subgroups 2 and 3, HL was the sole initial symptom for nearly all patients (92% and 100%, respectively). Across the cohort, 99 variants in 44 genes were identified, including 36 novel variants.

**Conclusion:**

The frequent absence of syndromic features at presentation may lead to genetic testing or analysis restricted to NSHL-associated genes. Our findings highlight the critical role of comprehensive genomic testing in the diagnostic workup of HL, enabling earlier identification of syndromic forms and facilitating timely medical management, genetic counseling, and anticipatory care.

**Supplementary Information:**

The online version contains supplementary material available at 10.1186/s10020-026-01431-6.

## Background

Hearing loss (HL) is one of the most common congenital conditions, affecting 1–2 in 1,000 newborns and 3–4 in 1,000 adolescents (Morton and Nance 2006). In developed countries, approximately 80% of congenital HL has a genetic basis (Vona et al. 2020). Adequate hearing is crucial for language and cognitive development and individuals with congenital HL often face challenges in these areas (Sajjad et al. 2008). HL exhibits enormous clinical and genetic diversity and is broadly classified as syndromic HL (SHL) or non-syndromic HL (NSHL). SHL accounts for approximately 20–30% of cases, while NSHL represents 70–80%. To date, more than 150 genes have been associated with NSHL and more than 600 syndromes include HL as a symptom (Parker and Bitner-Glindzicz 2015). Notably, the absence of additional symptoms in newborns or children does not necessarily exclude SHL, and an emerging appreciation of “non-syndromic mimics” is taking shape, whereby hearing impairment can be the first manifestation in a syndrome (Vona 2025). Studies estimate that approximately 20% of children initially clinically diagnosed with NSHL are later identified as having a syndrome (Downie et al. 2020; Sloan-Heggen et al. 2016). Early recognition of a syndromic etiology in individuals with apparently isolated HL is crucial, as it enables timely (pre-symptomatic) interventions, surveillance, and management of the associated medical complications.

Individuals with neurodevelopmental disorders (NDDs) have an increased risk of sensory impairments, including HL, which is more prevalent in this group than in the general population (Hey et al. 2014; Owrang and Vona 2025). SHL can be further categorized into syndromes with or without NDD involvement. NDDs themselves represent a genetically and clinically heterogeneous group of conditions, affecting 1–3% of children (Maulik et al. 2011), and include disorders such as intellectual disability, autism spectrum disorder, attention deficit hyperactivity disorder, and developmental delay. Interestingly, numerous NDD-associated syndromes may initially present with HL as the first diagnosed symptom, highlighting the diagnostic challenge posed by non-syndromic mimics (Owrang and Vona 2025). For example, *ANKRD11* is frequently associated with NDDs, with variants causing KBG syndrome (KBGS, MIM# 148050). KBGS is a rare genetic disorder characterized by distinctive craniofacial features, macrodontia of the upper central incisors, intellectual disability and short stature. Due to its subtle or mild features, KGBS can be underdiagnosed (Sirmaci et al. 2011), yet HL is reported in 27% of KBGS patients and can be the presenting symptom (Bianchi et al. 2017). Similarly, *KARS1* variants are associated with a spectrum of autosomal recessive disorders including NSHL (DFNB89, MIM# 613916), adult-onset progressive leukoencephalopathy with deafness (DEAPLE, MIM# 619196), infantile-onset progressive leukoencephalopathy with or without deafness (LEPID, MIM# 619147), as well as autism and hyperactivity (Lin et al. 2021). In many patients with *KARS1* variants, HL is the earliest clinical sign, with neurological symptoms appearing only later in adolescence or adulthood (Itoh et al. 2019; Scheidecker et al. 2019; Zhou et al. 2017; van der Knaap et al. 2019).

Among SHL cases without NDDs, Usher syndrome (USH, MIM# 276900) and Waardenburg syndrome (WS, MIM# 193500) are the most common. USH, the leading cause of combined hereditary hearing and vision impairment, is classified into four clinical subtypes (Bahena et al. 2022; Peter et al. 2021) with HL always appearing first. Deaf-blindness is a complex condition, as hearing and vision function together, with one sense compensating for the other through cross-modal plasticity (Bahena et al. 2022). As a result, deaf-blindness, which encompasses a broad spectrum of hearing and vision impairment (with 99% of patients retaining some degree of residual hearing and/or vision), is more complex than simply the combination of HL and vision impairment. Children with dual sensory impairments have unique educational requirements, necessitating a multidisciplinary approach involving a team of medical professionals (Dammeyer 2014). WS is another genetically and clinically heterogeneous disorder characterized by varying degrees of congenital HL and pigmentation abnormalities. Some subtypes, such as type 2E, as well as peripheral demyelinating neuropathy, central dysmyelination, Waardenburg syndrome, and Hirschsprung disease (PCWH MIM# 609136) may also cause central nervous system involvement, NDDs and muscle tone abnormalities (Inoue et al. 2004). In many of these conditions, HL often precedes other clinical signs and may be misdiagnosed as NSHL.

Advancements in next generation sequencing have significantly improved our ability to identify the genetic causes of HL, enabling precise diagnostics. However, when HL is the first symptom of a syndrome, initial genetic testing may be limited to NSHL-associated genes due to the absence of additional symptoms. The continuous expansion of genes classified as non-syndromic mimics further complicates diagnosis, delaying definitive diagnosis and appropriate early intervention.

We present clinical and molecular data from 111 patients with HL from 102 unrelated families. To identify the underlying molecular pathology and its relationship to clinical presentation, we performed molecular inversion probe panel, exome, or genome sequencing and classified patients into three groups based on variants and associated phenotypes. Our findings highlight the importance of comprehensive genomic testing and analysis beyond NSHL gene panels in patients with apparently isolated HL. Expanded genetic analysis can facilitate early diagnosis, enable timely intervention, and allow patients to participate in subsequent monitoring and treatment programs, ultimately improving long-term outcomes.

## Results

### Molecular genetic testing results

**Fig. 1:**
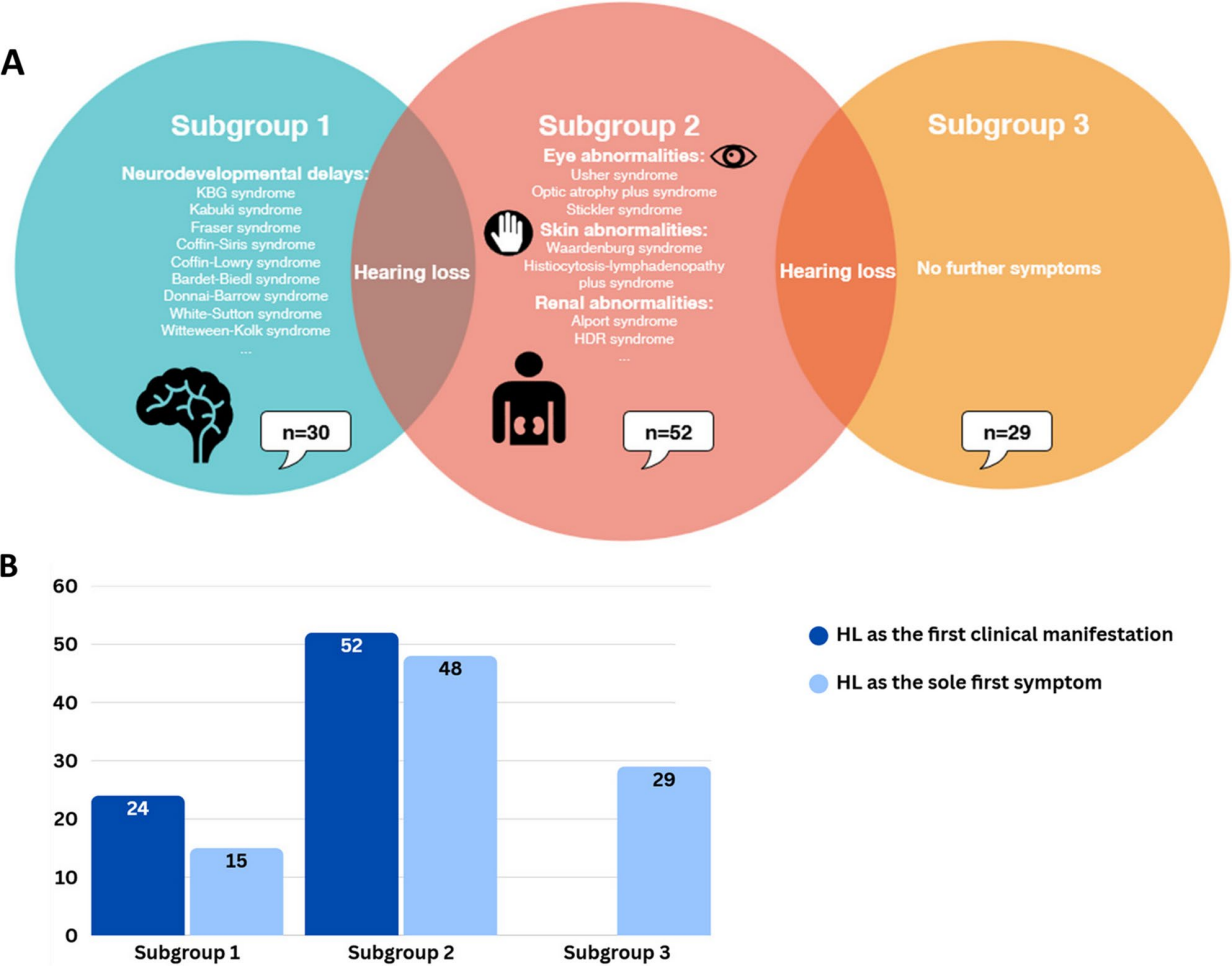
Overview of the phenotypic characteristics of our cohort. **A** The three subgroups presented in this study. **B** Clinical manifestation of HL in each subgroup. HL, hearing loss

**Fig. 2:**
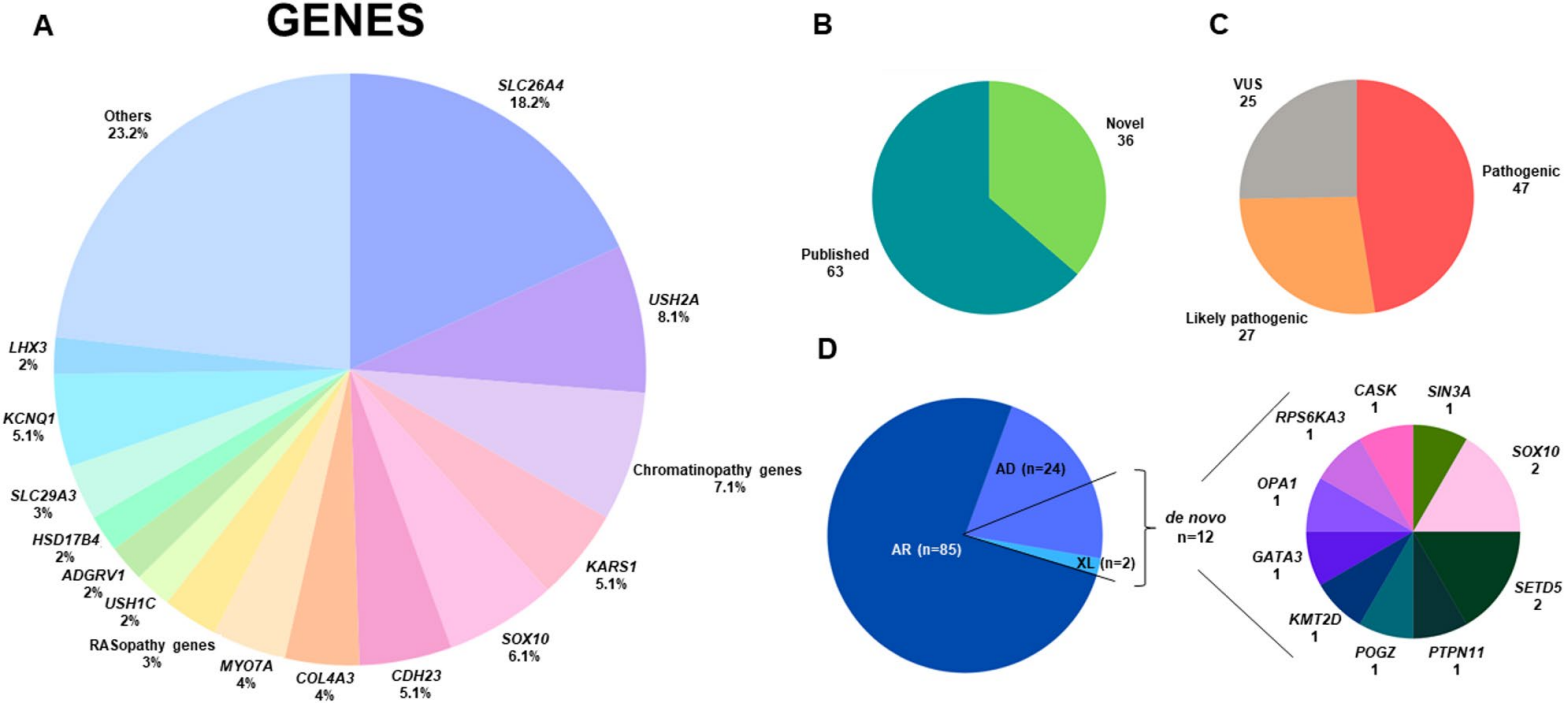
Overview of the genotypic characteristics of our cohort. **A** Genes with identified variants in the cohort. **B** Novel and published variants across the cohort. **C** Classification of identified variants across the cohort. **D** Inheritance patterns of the identified variants including genes with de novo variants

**Fig. 3:**
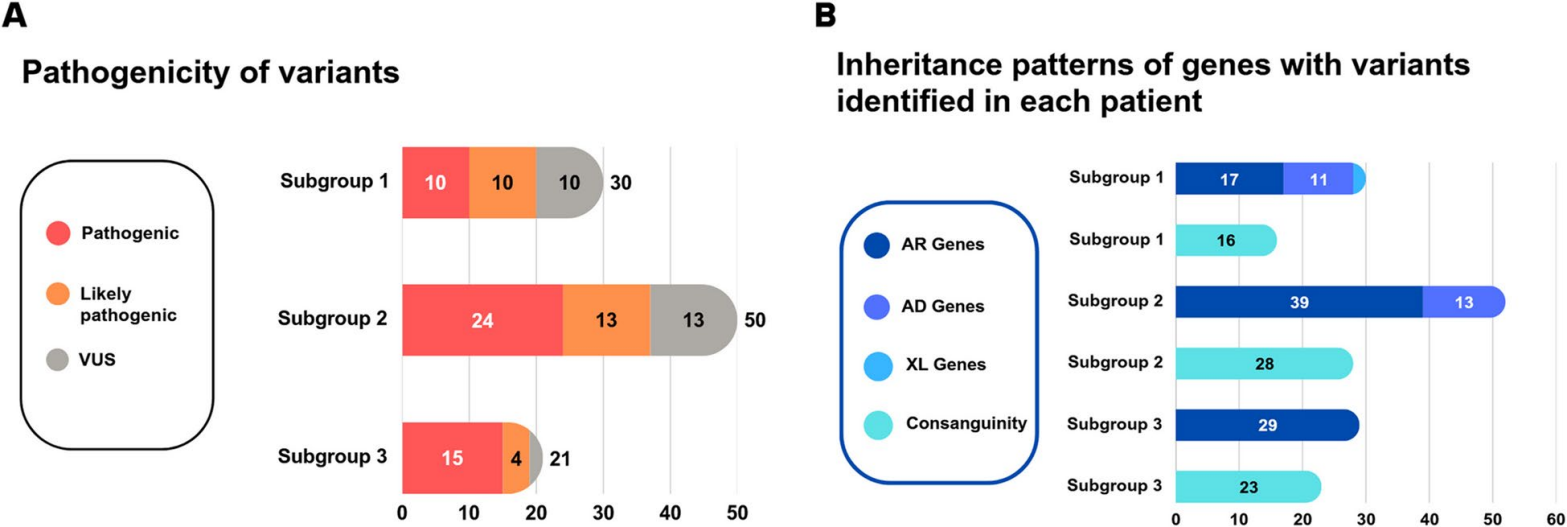
General overview of the characteristics of detected variants in each subgroup. **A** Pathogenicity of detected variants in subgroups 1–3 according to ACMG/AMP criteria. **B** Inheritance pattern of detected variants in subgroup 1–3. Turquoise columns indicate consanguineous families with variants in AR genes

One hundred eleven patients with HL from 102 distinct families included 31 patients with European ethnicity, 73 patients of Iranian descent, and 7 patients of Turkish descent were sequenced using a molecular inversion probe panel, exome, or genome sequencing approach. Following molecular sequencing and analysis, we classified patients into three subgroups based on the identified causative genes, not solely on clinical presentation (Fig. 1A). The first subgroup consists of 30 patients from 29 unrelated families with variants in NDD-associated genes. The second subgroup includes 52 cases from 46 unrelated families with variants in SHL genes not associated to NDDs. Finally, the third subgroup consists of 29 patients from 27 unrelated families with variants in genes associated with both SHL and NSHL, such as *SLC26A4* and *USH1C*. Ninety-nine variants in 44 genes were identified, including 36 novel variants (Fig. 2A, 2B). According to the updated recommendations for application of ACMG/AMP criteria, 47 variants were classified as pathogenic (P), 27 as likely pathogenic (LP), and 25 as variants of unknown significance (VUS) (Fig. 2C and 2A), all of which were missense. As a note, two P variants, c.415+2 T>C in *SLC26A4* and c.388-1G>C in *USH1C,* were listed in both subgroup 2 and subgroup 3, as one patient carrying the variant c.415+ 2T>C in *SLC26A4* exhibited features of Pendred syndrome, while the other two patients presented with NSHL and a patient with c.388-1G>C in *USH1C* presented Usher syndrome, while the other patient had NSHL. The ACMG/AMP criteria used for each variant, as well as indication of novel variants are shown in Table S1-3. Variants in genes associated with autosomal recessive disorders were identified in most cases (~ 77%, 85/111), which correlates with the high rate of consanguinity, present in ~ 66% (67/102) of the families (Fig. 2D and 3B). In addition, variants in genes associated with autosomal dominant disorders were identified in 24 patients, while variants in X-linked genes were detected in only 2 patients (Fig. 2D and 2B). Of the variants we identified in autosomal dominant genes, 10 were found to be de novo. All of the X-linked variants (*n* = 2) were also de novo (Fig. 2D). Unfortunately, we were not able to test the parents in all these families.

### Subgroup 1

In subgroup 1, most patients (80%, 24/30) exhibited HL as the first clinical symptom, while additional symptoms were identified in half (50%, 15/30) (Fig. 1B). We identified 30 different variants in 24 NDD-associated genes in 30 patients from 29 unrelated families. Table 1 shows both the clinical and genetic landscape of this subgroup. Of these, 10 each were variants classified as P, LP, and VUS (Fig. 3A, Table S1).

**Table 1:** Clinical and genetic landscape of Subgroup 1

ID	Gender	Age*	Gene	Variant	Zygosity	Clas	First clinical manifestation	HL-onset	HL-type	Further symptoms*	OMIM Phenotype
1	M	5y	*SIN3A* NM_001145358.2	c.3418C>T, p.(Arg1140*)	Het/de novo	P	HL	Pre-lingual	Bilateral, mild to moderate	NDD, diaphragmatic hernia, Pectus excavatum, clinodactyly V and dysmorphic facial features	Witteveen-Kolk syndrome, AD (MIM# 613406)
3	F	1y	*SOX10* NM_006941.4	c.698-4_698-2delinsATG	Het/NA	LP	HL	Congenital	Bilateral severe	NDD, no more details	PCWH syndrome, AD (MIM# 609136)
16	M	6y	*SOX10* NM_006941.4	c.376_377insG, p.(Tyr126*)	Het/de novo	LP	HL	Congenital	Bilateral, pan-frequency, profound	NDD	Waardenburg syndrome, type 4 C (MIM# 613266)
4	F	11y	*SETD5* NM_001080517.3	c.1333C>T, p.(Arg445*)	Het/de novo	P	VSD	Post-lingual, childhood	Bilateral, pan-frequency, moderate	HL at the age of 6 years, ADHD mild facial dysmorphism, mild ID, balance disorder	Intellectual developmental disorder, autosomal dominant 23, AD (MIM# 615761)
5	M	8y	*SETD5* NM_001080517.3	c.2182del, p.(Asp728Ilefs*9)	Het/de novo	P	AVSD, HL	Congenital	Unilateral, moderate	NDD, no more details	Intellectual developmental disorder, autosomal dominant 23, AD (MIM# 615761)
38	M	11y	*ANKRD11* NM_013275	c.6836_6837del, p.(Val2279Glyfs*16)	Het/NA	P	HL	Congenital	Bilateral, moderate	Dysmorphic facial features, short neck, puberte tarda, macrodontia, ptosis, hypoplastic cerebellar vermis, mild ID	KBG Syndrome, AD (MIM# 148050)
6	F	11y	*SMARCA4* NM_001128849.3	c.761G>T, p.(Gly254Val)	Het/NA	VUS	HL	Congenital	Bilateral, pan-frequency, severe	NDD, mild facial dysmorphism	Coffin-Siris syndrome 4, AD (MIM# 614609)
9	M	7y	*RPS6KA3* NM_004586.3	c.1741A>G, p.(Thr581Ala)	Hem/de novo	LP	HL	NA	NA	NDD, no more details	Coffin-Lowry syndrome, XLD (MIM# 303600)
109	M	NA	*PTPN11* NM_002834.3	c.5C>T, p.(Thr2Ile)	Het/de novo	LP	HL	Pre-lingual	NA	Facial dysmorphism, absent speech	Noonan syndrome 1, AD (MIM# 163950)
10	M	19y	*BRAF* NM_004333.6	c.2204G>A, p.(Arg735Gln)	Het/NA	VUS	HL	Pre-lingual	Bilateral, pan-frequency, moderate	ADHD, learning difficulties, inguinal hernia, mild dysmorphic facial features	Noonan syndrome 7, AD (MIM# 613706)
11	F	1y	*POGZ* NM_015100.4	c.2569A>G, p.(Arg857Gly)	Het/de novo	LP	Hypotonia, dysphagia microcephaly, ASD	Pre-lingual	Bilateral, moderate	HL, short stature, sleep apnea	White-Sutton syndrome, AD (MIM# 616364)
14	F	18y	*KARS1* NM_001130089.2	c.223del, p.(Gln75Serfs*2) c.1028T > C, p.(Val343Ala)	Comp het Comp het	P LP	HL	Congenital	Bilateral, sensorineural, profound	ID, adult-onset leukoencephalopathy, cerebellar ataxia and dystonia	DEAPLE, AR, (MIM# 619147)
66	M	5y	*KARS1* NM_001130089.2	c.379T>C, p.(Phe127Leu)	Hom	LP	HL, hypotonia	Congenital	Bilateral, profound	Microcephaly, severe NDD, spasticity, blindness, cMRI: leukodystrophy	Leukoencephalopathy, progressive, infantile onset, with or without deafness, AR (MIM# 619147)
67	F	5y	*KARS1* NM_001130089.2	c.889C>G, p.(Pro297Ala)	Hom	VUS	HL	Congenital	Bilateral, profound	NDD, poor balance and coordination	DEAPLE, AR, (MIM# 619147)
39	F	3y	*KARS1* NM_001130089.2	c.1124A>G, p.(Tyr375Cys)	Hom	VUS	HL, hypotonia	Congenital	Bilateral, profound, sensorineural	NDD, ataxia	Leukoencephalopathy, progressive, infantile onset, with or without deafness, AR (MIM# 619147)
71	M	7 m	*HSD17B4* NM_000414.4	c.394C>T p.(Arg132Trp)	Hom	LP	HL, hypotonia	Pre-lingual	NA	NDD, epilepsy	D-bifunctional protein deficiency, AR (MIM# 261515)
68	F	NA	*TWNK* NM_021830.3	c.874C>A, p.(Pro292Thr)	Hom	VUS	HL	Pre-lingual	NA	Ataxia	Perrault syndrome 5, AR (MIM# 616138)
24	F	8 m	*CASK* NM_003688.3	c.173-2331_278 + 1542del	Het/de novo	P	HL, microcephaly	Congenital	Bilateral, severe	Hypotonia	Intellectual developmental disorder and microcephaly with pontine and cerebellar hypoplasia, XLD (MIM# 300749)
60	M	15y	*BBS1* NM_024649.4	c.1642del, p.(Leu548Trpfs*31)	Hom	LP	Hypotonia, polydactyly	Pre-lingual	Mild to moderate	HL, ID, facial dysmorphism	Bardet-Biedl syndrome 1, AR (MIM# 209900)
62	F	,7y	*FRAS1* NM_025074.6	c.9607G>T, p.(Asp3203Tyr)	Hom	VUS	HL, cryptophthalmos, dysplastic ears, bifid nose, cleft palate and syndactyly	Congenital	Conductive, severe	Hypotonia, severe NDD, microcephaly	Fraser syndrome 1, AR (MIM# 219000)
63	M,	11y	*GALC* NM_000153.4	c.328+1G>T	Hom	P	Developmental regression	NA	NA	HL, mild microcephaly	Krabbe disease, AR (MIM# 245200)
64	M	5y	*PEX1* NM_000466.2	c.3116C>T, p.(Thr1039Ile)	Hom	VUS	HL	Pre-lingual	NA	NDD, blindness	Peroxisome biogenesis disorder 1 A and 1B, AR (MIM# 601539)
65	F,	3y (sister of 64)	*PEX1* NM_000466.2	c.3116C>T, p.(Thr1039Ile)	Hom	VUS	HL	Pre-lingual	NA	NDD, blindness	
69	M,	4y	*CACNA1D* NM_000720.3	c.1580G>A, p.(Arg527His)	Hom	VUS	HL, cleft palate, ASD	Congenital	NA	NDD recurrent seizures, facial dysmorphism, hypothyroidism, club foot, skin blisters	Sinoatrial node dysfunction and deafness, AR (MIM# 614896)
72	NA	NA	*LRP2* NM_004525.2	c.1342-2A>T	Hom	P	HL, diaphragmatic hernia	Congenital	NA	NDD	Donnai-Barrow Syndrome, AR (MIM# 222448)
74	M	2y	*ELOVL4* NM_022726.3	c.424A>G, p.(Thr142Ala)	Hom	VUS	HL	Congenital	NA	Ichthyosis, epilepsy, hypotonia	Ichthyosis, spastic quadriplegia, and impaired intellectual development, AR (MIM# 614457)
75	F	21y	*AAAS* NM_015665.5	c.398_399+2del, p.(Leu134Glnfs*8)	Hom	P	Alacrima	NA	NA	NDD, short stature, bilateral mild optic atrophy, HL, adrenocorticotropic hormone (ACTH)-resistant adrenal insufficiency, mineralocorticoid insufficiency, distal muscle weakness, atrophy	Triple-A syndrome, AR (MIM# 231550)
40	F	14y	*SGSH* NM_000199.5	c.364G>A, p.(Gly122Arg)	Hom	LP	HL	NA	NA	NDD, seizure, respiratory disorder, feeding difficulty	Mucopolysaccharidosis type IIIA, AR (MIM# 252900)
41	F	5y	*KMT2D* NM_003482.4	c.993dup, p.(Glu332Argfs*10)	Het/de novo	P	Cleft palate	NA	NA	HL, NDD, seizure, strabismus, facial dysmorphism	Kabuki syndrome 1, AD (MIM# 147920)
42	F	1y	*FGF3* NM_005247.4	c.166C>T, p.(Leu56Phe)	Hom	VUS	HL, microtia	Congenital	Bilateral, conductive, severe	No walking, no talking, no neck movement, hypotonia, NDD	Deafness, congenital with inner ear agenesis, microtia, and microdontia, AR (MIM# 610706)

^*^ at the last examination. *Abbreviations*: *Clas* classification, *F* female, *M* male, *y* years, *m* months, *P* pathogenic, *LP* likely pathogenic, *VUS* variant of unknown significance, *Het* heterozygous, *Hem* hemizygous, *Hom* homozygous, *Comp het* compound heterozygous, *NA* not available, *HL* hearing loss, *NDD* neurodevelopmental disorder, *VSD* ventricular septal defect, *ASVD* atrioventricular septal defect, *ASD* atrial septal defect, *ADHD* attention deficit hyperactivity disorder, *ID* intellectual disability, *AD* autosomal dominant, *AR* autosomal recessive

**Fig. 4:**
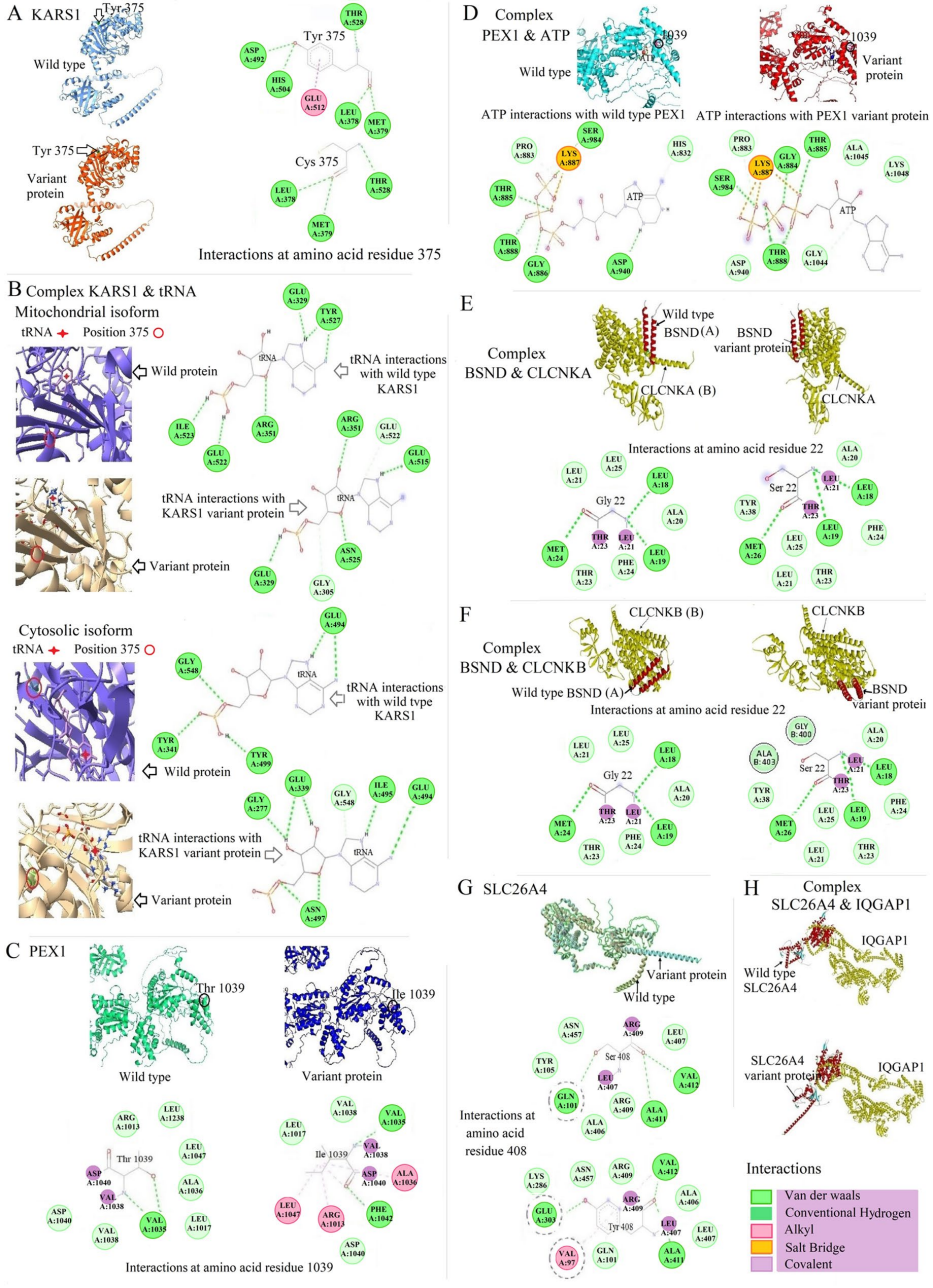
Molecular docking analysis of several variants classified as VUS. *KARS1* (NM_001130089.2) c.1124A>G, p.(Tyr375Cys)/(NM_005548.3) c.1040A>G, p.(Tyr347Cys) is shown in A-B; *PEX1* (NM_000466.2) c.3116C>T, p.(Thr1039Ile) is shown in C-D; *BSND* (NM_057176.3) c.64G>A, p.(Gly22Ser) is shown in E–F; and *SLC26A4* (NM_000441.1) c.1223C>A, p.(Ser408Tyr) is shown in G-H. **A** The 2D structure of the variant site's interaction in KARS1 (375 amino acid), indicating a reduction in the number of hydrogen bonds and decreased stability at the variant site. **B** Molecular docking analysis of the mitochondrial and cytosolic isoforms of the protein, along with tRNA, indicating a change in binding affinity. **C** The 2D structure of the PEX1 1039 amino acid interactions, indicating an increase in stability at the variant site. **D** Molecular docking analysis between ATP and PEX1, indicating an increase in binding affinity. **E** The interaction site of BSND with CLCNKA, indicating a global change in the binding site. (A) and (B) correspond to the protein being shown on the right. **F** The interaction site of BSND with CLCNKB, indicating a global change in the binding site. (A) and (B) correspond to the protein being shown on the right. **G** The lack of complete alignment of the wild-type with SLC26A4 p.(Ser408Tyr) highlights the importance of changes in the Ramachandran angles. Additionally, the 2D structure indicates changes in several bonds and the amino acids involved. H) The global change in the interaction site of SLC26A4 with IQGAP

*KARS1* variants (*n* = 5) were the most common in this subgroup. Patient 14 had compound heterozygous variants (c.223del, p.(Gln75Serfs*2) and c.1028T>C, p.(Val343Ala)), patient 66 presented with a homozygous variant (c.379T>C, p.(Phe127Leu)), patient 67 had a homozygous variant (c.889C>G, p.(Pro297Ala)), and patient 39 displayed a homozygous variant (c.1124A>G, p.(Tyr375Cys)). Three of these patients were included in a previous study (Lin et al. 2021) (Table S1). We constructed the predicted 3D structures of both the mitochondrial (c.1124A>G, p.(Tyr375Cys)) and cytosolic (c.1040A>G, p.(Tyr347Cys)) isoforms using SWISS-MODEL. These isoforms were aligned with PyMOL, which revealed no major local or global alterations. The substitution was identified at the terminal end of a helix, where a notable reduction in the number of hydrogen bonds was observed (Fig. 4A). It is important to note that hydrogen bonds are involved in the formation of secondary structures in macromolecules (Baker 2012). Furthermore, a published study (Wang et al. 2020) performed functional analysis of a neighbouring substitution (*KARS1* (NM_001130089.2) c.1129G>A, p.(Asp377Asn)) in both isoforms, revealing that alterations in the aminoacyl tRNA binding site were associated with HL. Considering proximity of the reported amino acid position to the mentioned site in our study, it is reasonable to propose that the subtle changes observed in the isoforms (Fig. 4B) due to the variant could be significant.

We also identified heterozygous variants in chromatinopathy-associated genes in seven patients: *ANKRD11* (c.6836_6837del, p.(Val2279Glyfs*16) in patient 38), *SETD5* (c.1333C>T, p.(Arg445*) in patient 4, c.2182del, p.(Asp728Ilefs*9) in patient 5), *KMT2D* (c.993dup, p.(Glu332Argfs*10) in patient 41), *SIN3A* (c.3418C>T, p.(Arg1140*) in patient 1), *POGZ* (c.2569A>G, p.(Arg857Gly) in patient 11) and *SMARCA4* (c.761G>T, p.(Gly254Val) in patient 6). Two patients exhibited variants in RASopathy genes, namely *PTPN11* (c.5C>T, p.(Thr2Ile) in patient 109) and *BRAF* (c.2204G>A, p.(Arg735Gln) in patient 10).

**Fig. 5:**
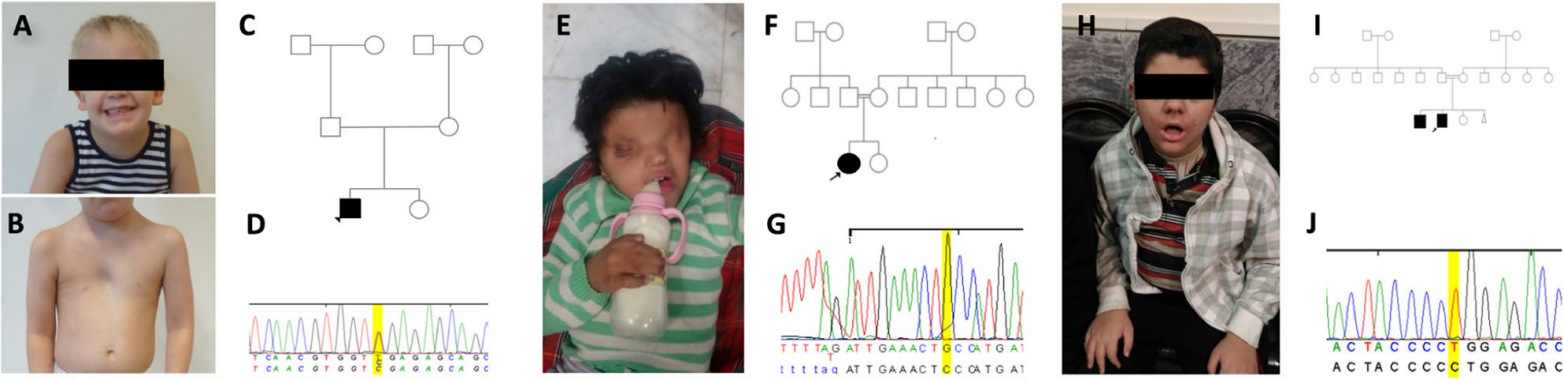
Selected patients from subgroup 1. **A**-**D **Patient 1 with Witteveen-Kolk syndrome. **A **Facial dysmorphism, of the patient 1, high forehead, epicanthal folds, smooth philtrum, long face, cupped ears, uplifted earlobes. **B **Pectus excavatum and webbed neck. **C **Pedigree of the patient (arrow head) with a heterozygous variant in *SIN3A* c.3418C>T, p.(Arg1140*). The parents are wild-type. **D **Sanger sequencing result for the de novo variant in *SIN3A* c.3418C>T, p.(Arg1140*) in patient 1. **E**–**G **Patient 62 with Fraser syndrome. **E **Facial dysmorphism of patient 62 showing plagiocephaly, hypertelorism, cryptophthalmos, dysplastic ears, and bifid nose. **F **Pedigree of the patient (arrow) with a homozygous variant in *FRAS1* c.9607G>T, p.(Asp3203Tyr). The parents are heterozygous carriers. **G **Sanger sequencing result for the homozygous variant in *FRAS1* c.9607G>T, p.(Asp3203Tyr) in the patient (arrow). **H**-**J **Patient 60 with Bardet-Biedl syndrome. **H **Facial dysmorphism of patient 60 showing a low anterior hairline with a widow peak, thick eyebrows with synophrys, telecanthus, bulbous nose, anteverted nares, marked thickening of the ala nasi, a protruding upper lip. **I **Pedigree of the patient (arrow) with a homozygous variant in *BBS1* c.1642del, p.(Leu548Trpfs*31). The parents are heterozygous carriers. **J **Sanger sequencing result for the variant in *BBS1* c.1642del, p.(Leu548Trpfs*31)

Interestingly, patient 1 initially presented with congenital HL and was diagnosed with NSHL until age three years and nine months. At two years and eight months, genetic testing for NSHL-associated genes yielded negative results. However, reanalysis at five years and 10 months revealed a de novo* SIN3A*, c.3418C>T, p.(Arg1140*) variant. Upon reevaluation, additional syndromic features were identified, including NDD, diaphragmatic hernia, pectus excavatum, clinodactyly V, and dysmorphic facial features such as high forehead, epicanthal folds, hypertelorism, downslanting palpebral fissures, smooth philtrum, long face, cupped ears, and uplifted earlobes (Fig. 5A-D).

We also examined patients with severe NDD. Patient 62, who presented with hypotonia, severe developmental delay, microcephaly, deafness, syndactyly and facial dysmorphism, which includes plagiocephaly, hypertelorism, cryptophthalmos, dysplastic ears, bifid nose and cleft palate, was identified with a homozygous variant c.9607G>T, p.(Asp3203Tyr) in *FRAS1*, leading to a diagnosis of Fraser syndrome 1 (MIM# 219000) (Fig. 5E-G).

Similarly, patient 60, who had intellectual disability, deafness, hypotonia, facial dysmorphism and polydactyly, was diagnosed with Bardet-Biedl syndrome type 1 (MIM# 209900) following identification of a homozygous variant in *BBS1* c.1642del, p.(Leu548Trpfs*31). Facial dysmorphism included a low anterior hairline with a widow’s peak, thick eyebrows with synophrys, telecanthus, bulbous nose, anteverted nares, marked thickening of the ala nasi, a protruding upper lip, and teeth anomalies (Fig. 5H-J).

Patients 64 and 65 are siblings with a medical history of NDD, HL and blindness, who were identified with a homozygous *PEX1* c.3116C>T, p.(Thr1039Ile) variant. This non-synonymous substitution is located at the terminal end of a helix in PEX1. The 2D structure of the variant was examined (Fig. 4C), which highlights the formation of a new hydrogen bond and the increase in the strength of a number of existing bonds. Since this protein utilizes ATP, its binding to ATP was evaluated, revealing no significant alterations in the binding site. However, changes in binding affinity are noted (Fig. 4D).

Additionally, we identified a structural variant in *CASK* through whole genome sequencing in patient 24. This heterozygous deletion c.173-2331_278+1542del spanned intron 2, exon 3 and intron 3. The patient referred at the age of 8 months due to HL and microcephaly, plagiocephaly, ptosis, broad nasal bridge and bulbous tip, large ears, hypotonia, overlapping toes, and hypoplastic toenails. The patient was diagnosed with intellectual developmental disorder with microcephaly and pontine and cerebellar hypoplasia (MICPCH, MIM# 300749).

### Subgroup 2

In subgroup 2, HL was the first symptom, with 92% (48/52) presenting HL as the sole initial symptom (Fig. 1B). Table 2 shows both the clinical and genetic landscape of this subgroup. We identified 50 different variants across 23 SHL genes in 52 patients from 46 unrelated families. Of these, 24 were classified as P, 13 as LP, and 13 as VUS, all of which were missense variants (Fig. 3A, Table 2). Table S3 presents the applied ACMG/AMP criteria for each variant.

**Table 2:** Clinical and genetic landscape of Subgroup 2

**ID**	**Gender **	Age*	**Gene**	**Variant**	**Zygosity**	**Clas**	**First clinical manifestation**	**HL-onset**	**HL-type**	Further symptoms*	**OMIM Phenotype**
2	M	5y	*GATA3* NM_001002295.2	c.1059delA, p.(Arg353Serfs*3)	Het/de novo	P	HL	Congenital	Bilateral, moderate, sensorineural, pan-frequency	No	HDR Syndrome, AD (MIM# 146255)
8	M	1y	*POLR1D* NM_015972.3	c.161C>A, p.(Ser54Tyr)	Het/paternal	VUS	HL	Congenital	Bilateral, moderate, mixed	Dysmorphic facial features	Treacher Collins syndrome 2, AD, AR (MIM# 613717)
21	F	32y	*USH2A* NM_015972.3	c.11864G>A, p.(Trp3955*)	Hom	P	HL	Congenital	Bilateral, sensorineural, severe	Retinitis pigmentosa	Usher syndrome, type 2 A, AR (MIM# 276901)
22	F	22y	*USH2A* NM_015972.3	c.653T>A, p.(Val218Glu) c.2299del, p.(Glu757Serfs*21)	Comp het Comp het	LP P	HL	Post-lingual	Bilateral, sensorineural, moderate	Retinitis pigmentosa	Usher syndrome, type 2 A, AR (MIM# 276901)
23	F	24y	*USH2A* NM_015972.3	c.920_923dup, p.(His308Glnfs*16) c.2299del, p.(Glu757Serfs*21)	Comp het Comp het	P P	HL	Post-lingual	Bilateral, sensorineural, moderate	Retinitis pigmentosa	Usher syndrome, type 2 A, AR (MIM# 276901)
13	M	40y	*USH2A* NM_206933.4	c.7595-2144A>G Exon 14 del	Comp het Comp het	P P	HL	Congenital	Bilateral, sensorineural, severe	Retinitis pigmentosa, cataract	Usher syndrome, type 2 A, AR (MIM# 276901)
28	F	67y	*USH2A* NM_206933.4	Exon 44 del	Hom	LP	HL	Post-lingual	Bilateral, sensorineural, severe	Retinitis pigmentosa	Usher syndrome, type 2 A, AR (MIM# 276901)
43	NA	NA	*USH2A* NM_206933.4	c.848+1G>T	Hom	LP	HL	NA	NA	NA	Usher syndrome, type 2 A, AR (MIM# 276901)
36	M	5y	*ADGRV1* NM_032119.3	c.12982G>T, p.(Glu4328*)	Hom	P	HL	Congenital	Bilateral, sensorineural, profound	No	Usher syndrome, type 2, AR (MIM# 605472)
37	F	37y	*ADGRV1* NM_032119.3	c.12982G>T, p.(Glu4328*)	Hom	P	HL	Congenital	Bilateral, sensorineural, profound	No	Usher syndrome, type 2, AR (MIM# 605472)
44	F	NA	*ADGRV1* NM_032119.3	c.4458T>G, p.(Tyr1486*)	Hom	P	HL	Congenital	Bilateral	No	Usher syndrome, type 2, AR (MIM# 605472)
45	M	6.5y	*MYO7A* NM_000260.3	c.4361T>G, p.(Val1454Gly) c.6231dup, p.(Lys2078Argfs*50)	Comp het Comp het	LP P	HL	Congenital	Bilateral, sensorineural, profound	Poor vision	Usher syndrome, type 1B, AR (MIM# 276900)
46	M	3y	*MYO7A* NM_000260.3	c.577A>C, p.(Thr193Pro)	Hom	VUS	HL	Congenital	Bilateral, sensorineural, moderate to severe	Motor DD, poor vision	Usher syndrome, type 1B, AR (MIM# 276900)
47	F	5y	*MYO7A* NM_000260.3	c.577A>C, p.(Thr193Pro)	Hom	VUS	HL	Congenital	Bilateral, sensorineural, profound	Motor DD, mild ID?	Usher syndrome, type 1B, AR (MIM# 276900)
106	F	34y	*MYO7A* NM_000260.3	c.1190C>A, p.(Ala397Asp)	Hom	LP	HL	Congenital	NA	Night blindness	Usher syndrome, type 1B, AR (MIM# 276900)
111	M	27 y (brother of P106)	*MYO7A* NM_000260.3	c.1190C>A, p.(Ala397Asp)	Hom	LP	HL	Congenital	NA	Night blindness, behavioral abnormalities	Usher syndrome, type 1B, AR (MIM# 276900)
112	F	3y (sister of P106)	*MYO7A* NM_000260.3	c.1190C>A, p.(Ala397Asp)	Hom	LP	HL	Congenital	NA	No	Usher syndrome, type 1B, AR (MIM# 276900)
48	F	7y	*CDH23* NM_022124.5	c.2349C>A, p.(Tyr783*)	Hom	P	HL	Congenital	Bilateral, sensorineural, profound	Motor DD	Usher syndrome, type 1D, AR (MIM# 601067)
49	M	7y	*CDH23* NM_022124.5	c.9040del, p.(Val3014*)	Hom	P	HL	Congenital	Bilateral, sensorineural, severe	Motor DD, seizure	Usher syndrome, type 1D, AR (MIM# 601067)
29	M	72y	*CDH23* NM_022124.5	Exons 4–6 del c.6050-70G>A	Comp het Comp het	P VUS	HL	Post-lingual	Bilateral, sensorineural, severe	Retinitis pigmentosa	Usher syndrome, type 1D, AR (MIM# 601067)
50	F	6y	*USH1C* NM_153676.3	c.388-1G>C	Hom	P	HL	Congenital	Bilateral, sensorineural, profound	Motor DD	Usher syndrome, type 1C, AR (MIM# 276904)
51	F	NA	*SOX10* NM_006941.3	c.373C>T, p.(Gln125*)	Het/de novo	P	HL	NA	NA	NA	Waardenburg syndrome, type 4C, AD (MIM# 613266)
52	F	NA	*SOX10* M_006941.3	c.137del, p.(Pro46Argfs*63)	Het/affected father	LP	HL	Congenital	Bilateral, sensorineural, severe, progressive	NA	Waardenburg syndrome, type 4C, AD (MIM# 613266)
53	F	NA	*SOX10* NM_006941.3	c.378C>G, p.(Tyr126*)	Het	P	HL	NA	NA	NA	Waardenburg syndrome, type 4C, AD (MIM# 613266)
110	M	NA	*SOX10* NM_006941.3	c.230del, p.(Ser77Thrfs*32)	Het	P	HL	Congenital	NA	Heterochromia	Waardenburg syndrome, type 4C, AD (MIM# 613266)
54	M	1y	*EDN3* NM_207034.2	c.472C>T, p.(Arg158Cys)	Hom	VUS	HL, heterochromia iridis	Congenital	Bilateral, profound, sensorineural	White forelock, Hirschsprung disease	Waardenburg syndrome, type 4B, AR (MIM# 613265)
19	M	9y	*HSD17B4* NM_000414.4	c.338A>T, p.(Asp113Val)	Hom	VUS	HL	Congenital	NA	NA	Perrault syndrome 1, AR (MIM# 233400)
20	F	4y (sister of P19)	*HSD17B4* NM_000414.4	c.338A>T, p.(Asp113Val)	Hom	VUS	HL	Congenital	NA	NA	Perrault syndrome 1, AR (MIM# 233400)
55	M	NA	*LARS2* NM_015340.4	c.1565C>A, p.(Thr522Asn)	Hom	LP	HL	Congenital	Bilateral, profound, sensorineural	NA	Perrault syndrome 4, AR (MIM# 604544)
61	M	2y	*OPA1* NM_130837.2	c.1819T>C, p.(Phe607Leu)	Het/de novo	LP	HL	NA	NA	Nystagmus	Optic atrophy plus syndrome, AD (MIM# 125250)
70	F	21y	*SLC29A3* NM_018344.5	c.1309G>A, p.(Gly437Arg)	Hom	LP	HL, heart anomaly	NA	NA	Wide sclerotic hyperpigmented hypertrichotic plaques	Histiocytosis-lymphadenopathy plus syndrome, AR (MIM# 602782)
56	M	3y	*SLC29A3* NM_018344.5	c.610+1G>A	Hom	P	HL	Congenital	Bilateral, sensorineural, severe	Cataract	Histiocytosis-lymphadenopathy plus syndrome, AR (MIM# 602782)
57	NA	NA	*SLC29A3* NM_018344.5	c.1087C>T, (p.Arg363Trp)	Hom	LP	HL	NA	NA	NA	Histiocytosis-lymphadenopathy plus syndrome, AR (MIM# 602782)
25	M	16y	*COL4A3* NM_000091.4	c.172G>A, p.(Gly58Ser)	Het	VUS	HL	Congenital	Bilateral, asymmetrical, mild to moderate	Hematuria	Alport syndrome 3A, AD (MIM# 104200)
73	F	11y	*COL4A3* NM_000091.4	c.4347_4353del, p.(Arg1450Valfs*77)	Hom	P	HL, failure to thrive	NA	NA	HL, short stature, epilepsy, anemia	Alport syndrome type 2, AR (MIM# 104200)
32	M	4y	*COL4A3* NM_000091.4	c.4862C>T, p.(Thr1621Met)	Hom	VUS	HL	Congenital	Bilateral, severe	Congenital hip dislocation, facial dysmorphism	Alport syndrome 3B, AR (MIM# 620536)
			*RAF1* NM_002880	c.1922C>T, p.(Thr641Met)	Het	VUS					Noonan Syndrome, AD (MIM# 611553)
33	F	1y (sister of 32)	*COL4A3* NM_000091.4	c.4862C>T, p.(Thr1621Met)	Hom	VUS	HL	Congenital	Bilateral, severe	Facial dysmorphism	Alport syndrome 3B, AR (MIM# 620536)
			*RAF1* NM_002880	c.1922C>T, p.(Thr641Met)	Het	VUS					Noonan Syndrome, AD, (MIM# 611553)
34	M	19y	*COL4A3* NM_000091.4	c.3882+5G>A	Hom	LP	HL	Post-lingual	Bilateral, sensorineural, moderate	Hematuria	Alport syndrome 3B, AR (MIM# 620536)
35	M	13y	*COL4A3* NM_000091.4	c.3882+5G>A	Hom	LP	HL	Post-lingual	Bilateral, sensorineural, moderate	Hematuria, proteinuria	Alport syndrome 3B, AR (MIM# 620536)
58	M	NA	*KCNQ1* NM_000218.2	c.514G>A, p.(Val172Met) c.877C>T, p.(Arg293Cys)	Comp het Comp het	VUS VUS	HL	NA	Bilateral	NA	Jervell and Lange-Nielsen syndrome, AR (MIM# 220400)
59	F	NA	*KCNQ1* NM_000218.2	c.912G>A, p.(Trp304*)	Hom	LP	HL	NA	Bilateral	NA	Jervell and Lange-Nielsen syndrome, AR (MIM# 220400)
76	NA	NA	*KCNQ1* NM_000218.2	c.683+1G>A	Hom	LP	HL	NA	Bilateral	Family history with sudden death	Jervell and Lange-Nielsen syndrome, AR (MIM# 220400)
77	NA	NA	*KCNQ1* NM_000218.2	c.1265dup, p.(Phe423Valfs*40)	Hom	P	HL	NA	Bilateral	NA	Jervell and Lange-Nielsen syndrome, AR (MIM# 220400)
78	M	1y	*BSND* NM_057176.3	c.64G>A, p.(Gly22Ser)	Hom	VUS	HL	Congenital	Bilateral, sensorineural, severe	Facial dysmorphism	Sensorineural deafness with mild renal dysfunction, AR (MIM# 602522)
79	M	6y	*SLC19A2* NM_006996.2	c.697C>T, p.(Gln233*)	Hom	P	HL	Congenital	Bilateral, sensorineural, severe	No	Thiamine-responsive megaloblastic anemia syndrome, AR (MIM# 249270)
80	M	5y	*LHX3* NM_014564.5	c.331G>A, p.(Ala111Thr) c.353T>G, p.(Leu118Arg)	Comp het Comp het	VUS VUS	HL	Congenital	Bilateral, sensorineural, profound	NA	Pituitary hormone deficiency, combined, 3, AR (MIM# 221750)
81	M	11y	*COL9A3* NM_001853.3	c.355del, p.(Leu119Serfs*10)	Hom	P	HL	Congenital	Bilateral, sensorineural, moderate to severe, progressive	Myopia, pes planus, depressed nasal bridge, anteverted nares, midface hypoplasia, down slanting palpebral fissures, X-Ray: spondyloepiphyseal dysplasia	Stickler syndrome, type VI, AR (MIM# 620022)
26	F	46y	*PAX3* NM_181458.4	c.808C>T, p.(Arg270Cys)	Het	P	HL, heterochromia iridis	Post-lingual	Unilateral, mixed, mild to m0derate	mild hypertelorism, telecanthus, multiple pigmentation disorders	Waardenburg syndrome 1, AD (MIM# 193500)
107	F	30y	*SLC26A4* NM_000441.1	c.415+2T>C	Hom	P	HL	Congenital	Bilateral, sensorineural, severe	Hypothyroid, diffuse thyroid enlargement	Pendred Syndrome, AR (MIM# 274600)
27	M	11y	*EYA1* NM_000503.6	c.639+98_760del, p.(Asp214Profs*112)	Het	P	HL	Post-lingual	Bilateral, asymmetrical, mixed, moderate to profound	Branchial cleft fistulas, preauricular fistulas	Branchiootic syndrome 1, AD (MIM# 602588)
30	F	2y sister 27	*EYA1* NM_000503.6	c.639+98_760del, p.(Asp214Profs*112)	Het	P	HL	Pre-lingual	Unilateral mixed, mild	Branchial cleft fistulas, preauricular fistulas	Branchiootic syndrome 1, AD (MIM# 602588)
31	F	36y mother 27	*EYA1* NM_000503.6	c.639+98_760del, p.(Asp214Profs*112)	Het	P	HL	Post-lingual	Bilateral, mixed, moderate	Branchial cleft fistulas, preauricular fistulas	Branchiootic syndrome 1, AD (MIM# 602588)

^*^ at the last examination *Abbreviations*: *Clas* classification, *F* female, *M* male, *y* years, *P* pathogenic, *LP* likely pathogenic, *VUS* variant of unknown significance, *Het* heterozygous, *Hem* hemizygous, *Hom* homozygous, *Comp het* compound heterozygous, *NA* not available, *HL* hearing loss, *DD* developmental delay, *ID* intellectual disability, *AD* autosomal dominant, *AR* autosomal recessive

Seventeen patients were diagnosed with USH, presenting biallelic variants in *USH2A*, *MYO7A*, *ADGRV1*, *CDH23*, and *USH1C.* Furthermore, we identified a heterozygous LP variant in *OPA1* in a patient with optic atrophy plus syndrome (MIM# 125250), a disorder associated with hereditary deaf-blindness.

**Fig. 6:**
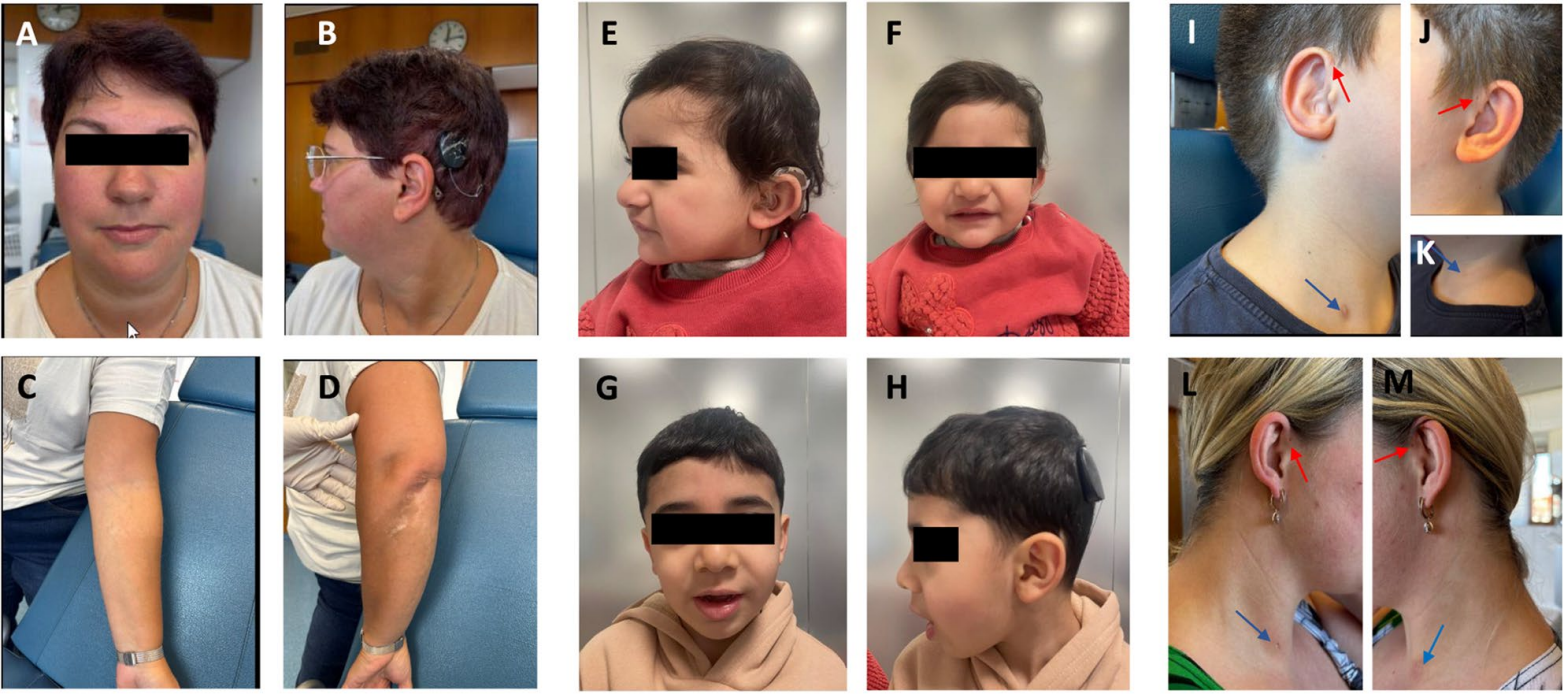
Selected patients from subgroup 2. **A**-**D **Patient 26 with Waardenburg syndrome was identified with a heterozygous variant in *PAX3* c.808C>T, p.(Arg270Cys). **A**-**B **Facial dysmorphism was present, including heterochromia iridis, hypertelorism, and telecanthus. **C**, **D **hypopigmented skin lesions. **E**, **F **Patient 33 and (**G**, **H**) patient 32 from the same family with a homozygous variant in *COL4A3* c.4862C>T, p.(Thr1621Met) and a heterozygous variant in *RAF1* c.1922C>T, p.(Thr641Met). **E**–**H **Craniofacial dysmorphism including downslanting palpebral fissures, hypertelorism, thick lips, and prominent chin in both siblings. **E **Low-set ears with thickened helix in patient 33. **I**-**K **Patient 27 with Waardenburg syndrome having a heterozygous variant in *EYA1* c.639+98_760del, p.(Asp214Profs*112). **I**-**K **with preauricular fistulas (red arrows) and branchial cleft fistulas (blue arrows). **L**, **M **Patient 31 (mother of patient 27) with preauricular fistulas (red arrows) and branchial cleft fistulas (blue arrows)

Six patients had variants in WS-associated genes, including *SOX10* (patients 51, 52, 53, and 110), *PAX3* (patient 26), and *EDN3* (patient 54). Patient 26 was initially diagnosed with NSHL and followed until the age of 46, at which point, a clinical geneticist noted heterochromia iridis, mild hypertelorism, telecanthus, and multiple pigmentation disorders (Fig. 6A-D). Notably, the patient’s dyed gray bangs were also observed during the medical history intake, raising suspicion of WS. Genetic testing revealed a heterozygous *PAX3* variant (c.808>T, p.(Arg270Cys)).

Interestingly, *COL4A3* variants were among the most common SHL-related variants in this subgroup, with four different variants in six patients (patients 25, 73, 32, 33, 34, and 35). In two siblings (patients 32 and 33), we identified a homozygous *COL4A3* variant (c.4862C>T, p.(Thr1621Met)), as well as a heterozygous *RAF1* variant (c.1922C>T, p.(Thr641Met)). Both siblings exhibited craniofacial dysmorphism, including downslanting palpebral fissures, hypertelorism, thick lips, and a prominent chin (Fig. 6E-H). Additionally, patient 33 had low-set ears with thickened helix (Fig. 6E-F).

Furthermore, we present a family with three affected individuals (patients 27, 30, 31). The index case was being followed in the otolaryngology clinic for NSHL. The DNA of this case was sent for genetic testing at the age of 11 years that revealed a novel pathogenic variant, c.639+98_760del, p.(Asp214Profs*112) in *EYA1*. Since *EYA1* is associated with SHL, reevaluation was recommended and the patient was diagnosed with Branchio-Otic Syndrome 1 (BOS1, OMIM #602588). Figures 5I–K show the branchial cleft fistulas and preauricular pits identified in the patient. The family history revealed that the mother and a sister also had HL. Subsequent clinical evaluation confirmed the diagnosis of BOS1 in both. Branchial cleft fistulas and preauricular pits were observed in the mother (Figs. 6L-M).

Patient 78 presented with a homozygous c.64G>A, p.(Gly22Ser) variant in *BSND*. The non-synonymous substitution is located within the transmembrane helix. Alignment of the wild-type and variant protein did not reveal major global changes. Molecular docking was performed to assess the variant impact of BSND, CLCNKA, and CLCNKB that showed significant changes in binding sites (Fig. 4E-F). Indeed, the binding site changed globally. The significant change in this in silico study highlights the importance of alterations in physicochemical properties and changes in the Ramachandran angles at the variant site in interaction with BSND.

### Subgroup 3

In subgroup 3, all patients (100%, 29/29) presented HL as the sole initial symptom. We examined 29 patients from 27 unrelated families, who were identified with variants in autosomal recessive SHL/NSHL genes, including *SLC26A4*, *USH1C* and *CDH23*. At the time of genomic testing, all patients had been clinically diagnosed with NSHL. Unfortunately, due to limited clinical data, we were unable to reevaluate half of these patients for unrecognized syndromic features.

We identified 21 different variants in three genes (*SLC26A4*, *CDH23*, *USH1C*), with 10 variants previously associated with SHL, including Pendred and Usher syndromes that are not clinically present in our patients. We also identified three novel variants (c.1028_1029insTCAG, p.(Ser344Glnfs*34) and c.1223C>A, p.(Ser408Tyr) in *SLC26A4* and c.388-1G>C in *USH1C*). While it is not possible to determine whether the two *SLC26A4* alleles are associated with SHL or NSHL, the *USH1C* variant c.388-1G>C was identified in a patient (50) from subgroup 2 of our cohort who exhibited Usher syndrome.

Patients 100, 114, 101, and 102 exhibited ophthalmologic abnormalities in addition to HL. While all four patients carried *SLC26A4* variants, this genotype alone does not account for the full clinical spectrum observed. These findings suggest either the involvement of additional, undetected genetic factors or a blended phenotype arising from dual diagnoses. Such cases highlight the diagnostic complexity inherent to SHL and the need for careful phenotypic and genetic evaluation.

The *SLC26A4* c.1223C>A, p.(Ser408Tyr) non-synonymous substitution is located at the end of an extracellular helix and causes a global change (Fig. 4G); however, this site does not exhibit direct interaction with IQGAP1 protein. Molecular docking of the variant was performed with IQGAP1 protein and showed substantial global changes (Fig. 4H). Table 3 shows both the clinical and genetic landscape of this subgroup. We identified 15 variants as P, 4 as LP and 2 as VUS (Fig. 3A, Table 3). Table S3 shows the literature research and applied ACMG/AMP criteria for each variant.

**Table 3:** Clinical and genetic landscape of Subgroup 3

ID	Gender	Age*	*Gene*	Variant	Zygosity	Clas	First clinical manifestation	HL-onset	HL-type	Further symptoms*	OMIM Phenotype
17	M	10y	*SLC26A4* NM_000441.1	c.1001+1G>A c.1334T>G, p.(Leu445Trp)	Comp het Comp het	P P	HL	Congenital	Bilateral, asymmetrical, mild to severe	NA	Pendred Syndrome, AR (MIM# 274600) Deafness, autosomal recessive 4, with enlarged vestibular aqueduct (MIM# 600791)
18	M	11y	*SLC26A4* NM_000441.1	c.919-2A>G	Hom	P	HL	Congenital	Bilateral, sensorineural, moderate to severe	NA	
82	M	NA	*SLC26A4* NM_000441.1	c.2027T>A, (p.Leu676Gln) c.578C>T, (p.The193Ile)	Comp het Comp het	P LP	HL	NA	NA	NA	
83	M	NA	*SLC26A4* NM_000441.1	c.1238A>G, p.(Gln413Arg)	Hom	P	HL	NA	Bilateral, sensorineural	NA	
84	F	NA	*SLC26A4* NM_000441.1	c.716T>A, p.(Val239Asp)	Hom	P	HL	NA	NA	NA	
85	F	39y	*SLC26A4* NM_000441.1	c.170C>A, p.(Ser57*)	Hom	P	HL	NA	NA	NA	
86	M	4y	*SLC26A4* NM_000441.1	c.716T>A, p.(Val239Asp)	Hom	P	HL	NA	NA	no	
87	M	19y	*SLC26A4* NM_000441.1	c.212T>A, p.(Ile71Asn) c.919-2A>G	Comp het Comp het	VUS P	HL	NA	Bilateral, sensorineural, severe to profound	NA	
88	M	4y	*SLC26A4* NM_000441.1	c.716T>A, p.(Val239Asp)	Hom	P	HL	Congenital	Bilateral, sensorineural	Kidney disease	
89	F	NA	*SLC26A4* NM_000441.1	c.716T>A, p.(Val239Asp)	Hom	P	HL	Congenital	Bilateral, sensorineural	no	
90	M	NA	*SLC26A4* NM_000441.1	c.716T>A, p.(Val239Asp)	Hom	P	HL	Congenital	Bilateral, sensorineural, mild to moderate	no	
108	F	51y	*SLC26A4* NM_000441.1	c.415+2T>C	Hom	P	HL	NA	NA	no	
91	M	22y	*SLC26A4* NM_000441.1	c.415+2T>C c.716T>A, p.(Val239Asp)	Hom	P P	HL	Congenital	NA	no	
92	F	NA	*SLC26A4* NM_000441.1	c.415+2T>C	Hom	P	HL	NA	NA	NA	
93	F	3y	*SLC26A4* NM_000441.1	c.704A>G, p.(Gln235Arg)	Hom	LP	HL	Congenital	Bilateral, sensorineural, severe	no	
113	M (brother of P93)	2y	*SLC26A4* NM_000441.1	c.704A>G, p.(Gln235Arg)	Hom	LP	HL	Congenital	Bilateral, sensorineural, severe	no	
94	M	4.5y	*SLC26A4* NM_000441.1	c.170C>A, p.(Ser57*)	Hom	P	HL	Congenital	Bilateral, sensorineural, moderate to severe	FMF	
95	M	10y	*SLC26A4* NM_000441.1	c.1223C>A, p.(Ser408Tyr)	Hom	VUS	HL	Pre-lingual	NA	no	
96	M	NA	*SLC26A4* NM_000441.1	c.2106del, p.(Lys702Asnfs*19)	Hom	P	HL	Congenital	Bilateral	NA	
97	M	NA	*SLC26A4* NM_000441.1	c.2106del, p.(Lys702Asnfs*19)	Hom	P	HL	Congenital	Bilateral	NA	
98	F	NA	*SLC26A4* NM_000441.1	c.845G>A, p.(Cys282Tyr) c.1028_1029insTCAG, p.(Ser344Glnfs*34)	Comp het Comp het	LP P	HL	Congenital	Bilateral, sensorineural	NA	
99	M	NA	*SLC26A4* NM_000441.1	c.1001G>T, p.(Gly334Val)	Hom	P	HL	NA	NA	NA	
100	F	NA	*SLC26A4* NM_000441.1	c.1234G>T, p.(Val412Phe)	Hom	LP	HL	Congenital	Bilateral, sensorineural	Visual problems	
114	F (sister of P100)	NA	*SLC26A4* NM_000441.1	c.1234G>T, p.(Val412Phe)	Hom	LP	HL	Congenital	Bilateral, sensorineural	Visual problems	
101	M	36y	*SLC26A4* NM_000441.1	c.1198del, p.(Cys400Valfs*32)	Hom	P	HL	Congenital	Bilateral, sensorineural	Myopia, vertigo since age 30y	
102	F	24y	*SLC26A4* NM_000441.1	c.1334T>G, p.(Leu445Trp)	Hom	P	HL	Congenital	Bilateral, sensorineural	Myopia	
105	M	13y	*CDH23* NM_022124.5	c.2398-1G>T	Hom	P	HL	NA	NA	NA	Deafness, autosomal recessive 12 (MIM# 601386) Usher syndrome, type 1D, AR and type 1D/F (MIM# 601067)
103	F	1y	*USH1C* NM_153676	c.463C>T, p.(Arg155*)	Hom	P	HL	Congenital	Bilateral, sensorineural	NA	Deafness, autosomal recessive 18A (MIM# 602092) Usher syndrome, type 1C, AR (MIM# 276904)
104	M	4y	*USH1C* NM_153676.3	c.388-1G>C	Hom	VUS	HL	Congenital	Bilateral, sensorineural, profound	NA	

^*^at the last examination *Abbreviations*: *Clas* classification, *F* female, *M* male, *y* years, *P* pathogenic, *LP* likely pathogenic, *VUS* variant of unknown significance, *Het* heterozygous, *Hom* homozygous, *Comp het* compound heterozygous, *NA* not available, *HL* hearing loss, *FMF* Familial Mediterranean fever, *AD* autosomal dominant, *AR* autosomal recessive

## Discussion

NDDs refer to conditions in which a child's nervous system is not fully developed and can cause delays in speech, social interaction, emotional regulation, behavior, motor skills, and cognition, ultimately affecting both psychological and physical health. These disorders can lead to long-term chronic conditions and disabilities into adulthood. Early identification and intervention can significantly improve long-term outcomes, with developmental screening and early intervention programs playing a crucial role in providing necessary support and therapies (Grantham-McGregor et al. 2007; Aldharman et al. 2023). In the first subgroup, 80% (24/30) of patients presented HL as the first clinical manifestation, with it being the sole first symptom in half (Fig. 1B). Among the NDD-associated SHL genes, *KARS1* was the most frequently affected with four patients reporting variable clinical impact. One particularly striking case was patient 14, who was followed as having NSHL until the age of 16. However, after neurological symptoms emerged and rapidly progressed, genetic testing revealed a diagnosis of DEAPLE. Currently, the patient is in intensive care, and the family is devastated by the unexpected findings. Had an earlier diagnosis been made and the family provided with appropriate genetic counseling; they could have been better prepared for this outcome.

Seven patients from subgroup 1 had variants in chromatinopathy-associated genes, including *SETD5* (*n* = 2), *ANKRD11* (*n* = 1), *KMT2D* (*n* = 1), *SIN3A* (*n* = 1), *POGZ* (*n* = 1) and *SMARCA4* (*n* = 1). Chromatinopathies are a class of NDDs caused by variants affecting chromatin regulators, encompassing over 60 disorders (Squeo et al. 2020). Among these, *ANKRD11* is one of the most frequently mutated genes associated with KBGS that can be underdiagnosed due to mild clinical features (Sirmaci et al. 2011). SET domain-containing 5 (SETD5) is a chromatin regulator and lysine methyltransferase and another common cause of neurodevelopmental disorders (intellectual developmental disorder, autosomal dominant 23 syndrome (MRD23, MIM# 615761)). Patients with *SETD5* and *ANKRD11* P variants exhibit overlapping clinical features (Crippa et al. 2020). Interestingly, while HL has been reported in some clinically diagnosed KBGS patients, it has not been identified as a symptom in patients with *SETD5* variants so far (Gnazzo et al. 2020). Patients 41, 1, 11, and 6 with Kabuki syndrome 1 (KS, MIM# 147920), Witteveen-Kolk syndrome (WITKOS, MIM# 613406), White-Sutton syndrome (WHSUS, MIM# 616364), and Coffin-Siris syndrome 4 (CSS4, MIM# 614609), caused by variants in *KMT2D*, *SIN3A*, *POGZ*, and *SMARCA4*, respectively, exhibit HL as one of the clinical manifestations. Up to 50% of patients with KS (Adam and Hannibal 1993) and CSS (Schrier Vergano et al. 1993) have HL that is the main symptom due to their mild clinical features, especially when an abnormality is detected during newborn hearing screening (Qiu and Yuan 2019; Kosho, Okamoto, and Coffin-Siris Syndrome International 2014). Some individuals with WITKOS and WHSUS have HL (Balasubramanian et al. 2021). Patient 1 with WITKOS having a c.3418C>T, p.(Arg1140*) variant in *SIN3A* was misdiagnosed with NSHL until age three years and nine months. Molecular testing led to the recognition of further previously unrecognized symptoms. In contrast to patient 1, neurological symptoms were the first to be identified in patient 11 with WHSUS, followed by the detection of HL. Five out of the seven patients with chromatinopathy-associated variants were initially tested due to HL, with syndromic features only recognized later.

In subgroup 1, 17 of 30 patients were found to have P variants in genes with an autosomal recessive inheritance pattern, with carrier status confirmed in their parents. Consequently, a prenatal diagnosis or preimplantation genetic testing could have been an option for these families—particularly those with a severely affected child or a child with a poor prognosis (Sullivan-Pyke and Dokras 2018).

In the second subgroup, almost all patients (92% 48/52) presented with HL as the sole initial symptom (Fig. 1B). Notably, 18 patients (35%) have variants identified in genes associated with deaf-blindness. We identified a LP c.1190C>A, p.(Ala397Asp) variant in *MYO7A* in three members of a family (patients 106, 111 and 112) within this group. Patients 106 and 111, aged 34 and 27, respectively, exhibited night blindness consistent with USH, while their 3-year-old sibling (patient 112) has so far presented isolated HL. Considering that late-onset vision loss was observed in the two older siblings after the age of 20, it is likely that the younger child may also develop USH later in life. Therefore, clinical follow-up has been recommended for this family.

Six individuals from four unrelated families (patient 25, 73, 32, 33, 34, 35) were identified with *COL4A3* variants*.* These variants are associated Alport syndrome (AS) which can be transmitted in an autosomal dominant or recessive pattern (Fallerini et al. 2014). *COL4A3* associated AS is characterized by kidney involvement, often accompanied by sensorineural HL, and sometimes by ocular abnormalities (Storey et al. 2013). The autosomal recessive form of AS typically results in more severe disease manifestations, whereas the autosomal dominant form progresses more slowly, with renal insufficiency and sensorineural HL developing later in life (Fallerini et al. 2014). Five patients with homozygous variants experienced more severe symptoms than patient 25 who had a heterozygous variant.

Additionally, five truncating variants in *SOX10* were identified in five unrelated patients, leading to different outcomes. Three individuals, who showed no neurological involvement, were clinically diagnosed with WS 4 C and had loss-of-function variants predicted to undergo nonsense-mediated decay (NMD). However, patient 3, with severe neurological symptoms, was identified with another truncating variant in *SOX10* that is predicted to escape NMD and placed in subgroup 1 and could be diagnosed with PCWH. Literature suggests that mutant mRNAs escaping NMD are associated with more severe disease phenotypes (Inoue et al. 2004). In addition, variants were identified in other WS associated genes, namely *EDN3* and *PAX3*. Patient 26, initially diagnosed with NSHL and followed until the age of 46, was later diagnosed with WS 1 due to a *PAX3* variant.

During this study, we identified five biallelic variants in *KCNQ1* in four unrelated patients (Table 2). The variants are consistent with a molecular diagnosis of Jervell and Lange-Nielsen syndrome (JLNS, MIM# 220400), a rare autosomal recessive disorder characterized by congenital sensorineural HL and a markedly prolonged QT interval, which predisposes affected individuals to malignant arrhythmias and sudden cardiac death (Schwartz et al. 2012). The presence of biallelic LP/P *KCNQ1* variants in these patients confirms the genetic etiology of their HL and has immediate implications for clinical management. Standard care for JLNS includes auditory rehabilitation (e.g., cochlear implants), β-blocker therapy, and in high-risk cases, consideration of implantable cardioverter-defibrillator implantation to prevent sudden death (Giudicessi and Ackerman 2013). Of note is patient 76 with a LP c.683+1G >A, who had family history of sudden cardiac death, further supporting the clinical suspicion of JLNS prior to genetic confirmation. Identification of causative *KCNQ1* variants not only enables tailored treatment strategies but also facilitates cascade genetic testing and counseling for at-risk family members.

Another set of findings in this subgroup involved five patients (patients 68 and 71 from subgroup 1 and patients 19, 20 and 55 from subgroup 2) who were diagnosed with Perrault syndrome (PS) due to variants in *HSD17B4*, *TWNK* and *LARS2*. PS is characterized by prelingual, progressive sensorineural HL, which may appear in early childhood. In some individuals, neurological symptoms emerge later, including learning difficulties, developmental delay, cerebellar ataxia, and motor and sensory peripheral neuropathy (Newman et al. 1993). Among the affected individuals, patients 68 and 71 had already exhibited neurological involvement at the time of their evaluations.

A particularly noteworthy aspect of subgroup 2 is that, at the time of their latest examinations, 20 patients exhibited no additional symptoms beyond HL. However, following their genetic diagnosis, these patients and their families were informed about potential future symptoms, enabling early intervention, when necessary. This proactive genetic counseling plays a crucial role in allowing families to anticipate medical complications, seek appropriate treatments, and, in some cases, implement pre-symptomatic interventions.

In subgroup 3, HL was the initially detected symptom in 29 (100%) patients in the third subgroup (Fig. 1B). These patients had a clinical diagnosis of NSHL with 18 different *SLC26A4* variants. Among these, eight (c.1001+1G>A; c.1334T>G, p.(Leu445Trp); c.919-2A>G; c.2027T>A, p.(Leu676Gln); c.1238A>G, p.(Gln413Arg); c.716T>A, p.(Val239Asp); c.212T>A, p.(Ile71Asn); c.704A>G, p.(Gln235Arg)) were previously reported in patients diagnosed with Pendred syndrome (MIM# 274600) (Zhao et al. 2024; de Moraes et al. 2016; Walsh et al. 2006; Tesolin et al. 2021; Smits et al. 2022). Interestingly, the c.415+2T>C variant in *SLC26A4* was identified in three patients from unrelated families. One of these patients (patient 108) belongs to subgroup 2 and presents symptoms consistent with Pendred syndrome, whereas the other patients (patients 107 and 92) fall into subgroup 3 and, exhibited only HL.

Furthermore, we detected three pathogenic variants in *CDH23*, and *USH1C* in three patients in subgroup 3. At least two (c.463C>T, p.(Arg155*) in *USH1C* and c.2398-1G>T in *CDH23*) of these variants have been linked to USH or a retinal disease in the literature (Huang et al. 2015; Bonnet et al. 2016; Ellingford et al. 2016).

Our genetic testing strategy included targeted exome sequencing for almost all patients, gene panel testing in some cases, and WGS in selected individuals with syndromic features or unclear molecular diagnoses. While WGS offers broader coverage, including non-coding regions and structural variants, it still has limitations. Some variants may be missed due to current annotation limitations, incomplete understanding of regulatory elements, or low-level mosaicism. For patients 100, 114, 101, and 102, who carried *SLC26A4* variants but also presented with ophthalmologic abnormalities, no additional clearly pathogenic variants were identified through the testing performed. These findings raise the possibility of complex inheritance patterns, blended phenotypes involving multiple genetic conditions, or the involvement of novel genes not yet linked to HL. Future application of WGS or other advanced genomic approaches in such cases may help clarify the underlying molecular diagnoses. Together, these cases underscore the ongoing challenges in achieving a complete understanding of SHL, even with comprehensive genetic testing.

In this cohort, 99 variants across 44 genes were identified, including 36 novel variants (Fig. 2, Table S1–S3), thereby expanding the known genotypic and phenotypic spectrum of SHL. Of these, 24 variants affected autosomal dominant and two in X-linked syndromic genes, yielding a total of 26 variants. Among these, 12 (46%) were determined to be de novo. Although this proportion is somewhat lower than the ~ 65% reported in the literature (Klimara et al. 2022), it is important to acknowledge that parental testing could not be conducted in some families within the cohort, which is a study limitation. An additional limitation of the study is the high rate of consanguinity, particularly among families of Iranian descent. It is essential to note that in the molecular docking studies, only the most stable positions before and after the substitutions were examined. However, it should be acknowledged that molecules in 3D space possess kinetic energy and exhibit movement; therefore, the evaluations conducted are estimations with these limitations.

## Conclusion

**Fig. 7:**
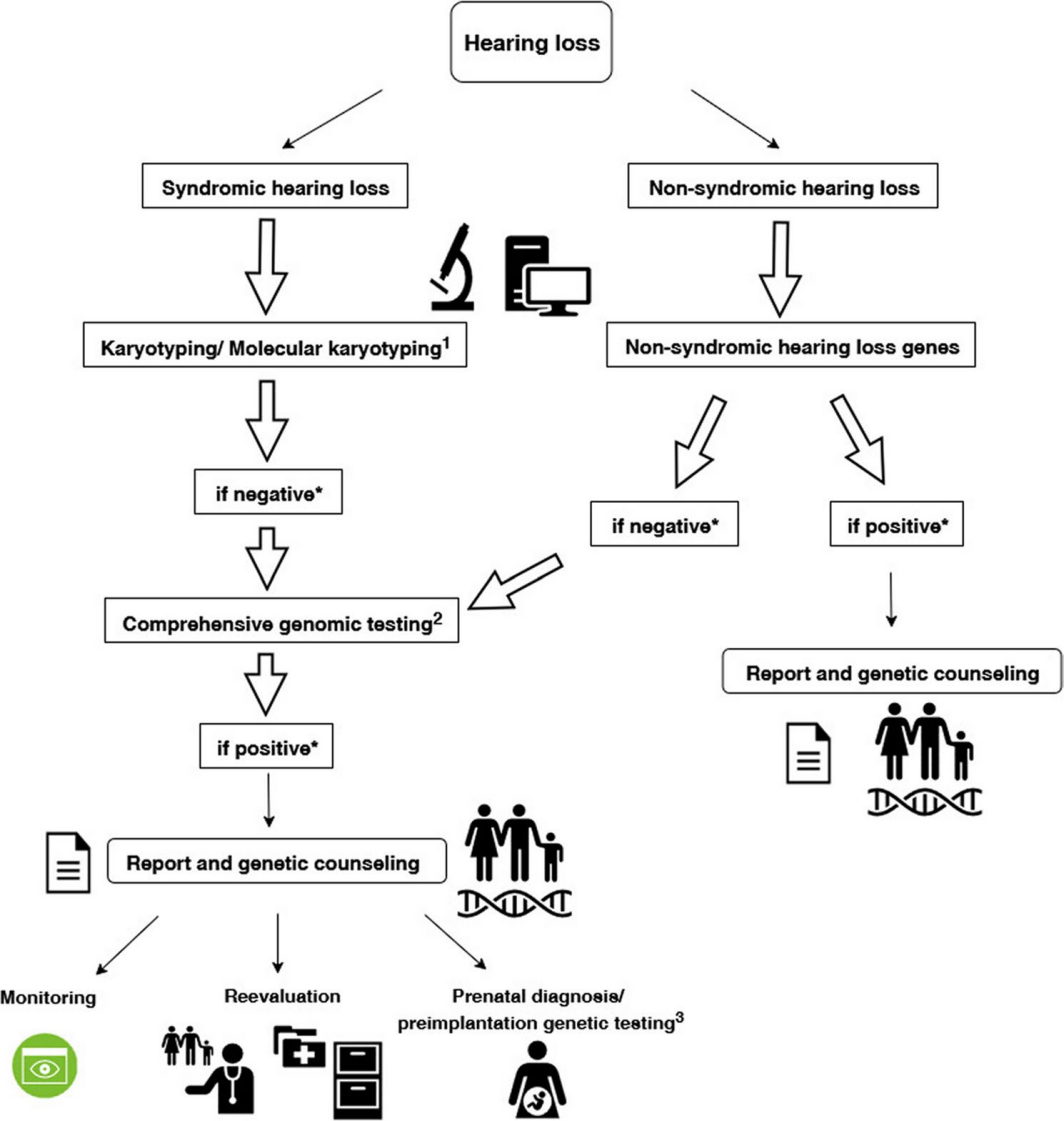
Flow diagram showing the clinical and diagnostic evaluation of patients with syndromic and non-syndromic hearing loss, respectively. ^1^: for patients with neurodevelopmental delays; ^2^: whole-exome or whole-genome sequencing; ^3^:for families with confirmed carrier status in parents who have a severely affected child or a child with a poor prognosis; *: "Positive" generally refers to likely pathogenic and pathogenic variants. In some cases, it may also include VUS + variants, particularly missense variants, that strongly support the patient’s clinical phenotype, even though they do not meet the full criteria for classification as likely pathogenic under ACMG guidelines. "Negative" generally refers to cases where no causative variants were identified, including those with benign, likely benign, or weak VUS that are not supportive of the clinical phenotype

Our study emphasizes that the initial clinical presentation of HL, even in the absence of additional symptoms, does not exclude syndromic forms. The identification of pathogenic variants in SHL genes associated with or without NDDs in patients who initially presented solely with HL underscores the diagnostic value of broad genetic testing. Based on these findings, we developed a schematic representation in Fig. 7 illustrating the recommended approach to patients with SHL and NSHL. Comprehensive genomic analysis, beyond NSHL-associated genes, enables earlier and more accurate diagnosis, facilitates anticipatory management of syndromic features, and provides critical information for genetic counseling.

## Methods

### Patient enrolment and ethical considerations

This study was approved by the ethics committees at the Medical Faculty of the University of Würzburg (approval numbers 131/21, 226/18 and 46/15), the University of Tübingen (approval number 197/2019BO01) and the University Medical Center Göttingen (approval number 3/2/16). Families were ascertained in Germany, as well as part of a large ethnically diverse Iranian and Turkish population rare disease study. From an initial pool of over 600 individuals with HL who were negative for *GJB2* and *STRC* variants/copy number variations, we selected, post hoc to genetic testing, cases in which genetic testing revealed a variant that either contraindicated or left open the possibility of revising the initial clinical diagnosis of NSHL. This targeted selection enabled focused analysis of patients whose findings suggested a potential syndromic etiology. Based on subsequent clinical and genetic analysis, patients were stratified into three sub-groups. Audiometry and medical information were collected from participating individuals for evaluation.

### Genetic and phenotypic analysis

Blood samples were collected from all participants after obtaining informed consent from patients or their parents. Informed consent from the parents or legal guardians of the patients/participants was obtained for the publication of their data. Genomic DNA was extracted using standard methods. Sequencing methods, data analysis and filtering strategy summaries per individual sequenced are summarized in Table S4. Gene content of the 89-gene molecular inversion probe panel is included in Table S5.

Standard clinical examinations including physical examination, and potentially diagnostic tests or investigations, such as ophthalmic examination, cMRI, and IQ-Test were also performed. Severity of HL was determined most commonly using pure-tone audiometry or other age-appropriate methods. Severity of HL was determined by calculating pure tone averages of frequencies between 0.5 to 4 kHz (PTA_0.5-4kHz_) following established guidelines to determine the severity of HL as mild (21–40 dB), moderate (41–70 dB), severe (71–95 dB), or profound (> 95 dB) according to established guidelines (Mazzoli et al. 2003). Pure-tone audiometry hearing thresholds were measured at frequencies of 0.25, 0.5, 1, 2, 4, and 8 kHz.

### Variant classification

Variant classification followed the ACMG/AMP criteria (Richards et al. 2015). In addition, the updated criteria from the following publications were used: ClinGen Sequence Variant Interpretation Recommendation for application of PM2 and PM3, Abou Tayoun et al. 2018 (Abou Tayoun et al. 2018) for application of PVS1, Brnich et al., 2020 (Brnich et al. 2019) for application of PS3**,** Pejaver et al. 2022 (Pejaver et al. 2022) for missense variant classification, Walker et al. 2023 (Walker et al. 2023) for splice variant classification and ClinGen Hearing Loss Expert Panel Specifications to the ACMG/AMP Variant Interpretation Guidelines for *CDH23*, *MYO7A*, *SLC26A4*, and *USH2A*. VUS were retained and reported in this study. To assess evidence for the interpretation of variants, in silico protein prediction tools such as AlphaMissense (Cheng et al. 2023), CADD (Rentzsch et al. 2019), and REVEL (Ioannidis et al. 2016) and splicing prediction program SpliceAI (Jaganathan et al. 2019), as well as ClinVar, Deafness Variation Database, HGMD and LitVar2 databases were used. Population databases like gnomAD v4.1.0, GME Variome Project, and Iranome revealed the population frequency of a given variant in a heterozygous or homozygous state.

### Variant validation and segregation analysis

Sanger sequencing, multiplex ligation-dependent probe amplification, and quantitative PCR were used to confirm variants and their segregation patterns across available family members. Bidirectional Sanger sequencing of PCR products was performed using standard protocols. Chromas version 2.6.6 and Alamut Visual Plus version 1.12 were used to analyze DNA sequences.

### Molecular docking

Initially, the three-dimensional (3D) structure of the wild-type KARS1, PEX1, BSND, and SLC26A4 proteins were searched in the Protein Data Bank (https://www.rcsb.org/) or UniProt (https://www.uniprot.org/). If available, the structure was downloaded at the lowest resolution. In cases where the PDB structure was not found in these databases, it was sought in the AlphaFold platform (https://deepmind.google/technologies/alphafold/). If a suitable structure was identified, the prediction score for the variant site and its surrounding region was evaluated. Upon obtaining a satisfactory prediction, the corresponding PDB file was downloaded.

The 3D structure of the variant protein was constructed and downloaded using SWISS-MODEL (https://swissmodel.expasy.org/). Subsequently, alignment of the wild-type and variant proteins (KARS1 p.(Tyr375Cys)/(Tyr347Cys), PEX1 p.(Thr1039Ile), BSND p.(Gly22Ser), and SLC26A4 p.Ser408Tyr) was performed using PyMOL (https://www.pymol.org/) and Chimera (https://www.cgl.ucsf.edu/chimera/) software to detect global changes. The structure was further investigated using the BIOVIA Discovery Studio Visualizer (https://www.3ds.com/products/biovia/discovery-studio/visualization).

Following this, a review of reported interactions in UniProt, along with relevant literature and other databases, was conducted to identify several key proteins or small molecules with potential interactions for molecular docking studies. Molecular docking between macromolecules was carried out using HDOCK (http://hdock.phys.hust.edu.cn/), while docking between macromolecules and small molecules was performed using PyRx (https://pyrx.sourceforge.io/). The results were analyzed using Discovery Studio Visualizer.

### Web resources

https://clinicalgenome.org/working-groups/sequence-variant-interpretation/.

https://www.clinicalgenome.org/docs/clingen-hearing-loss-expert-panel-specifications-to-the-acmg-amp-variant-interpretation-guidelines/.

https://www.omim.org/.

https://gnomad.broadinstitute.org/.

https://varsome.com/.

https://www.ncbi.nlm.nih.gov/clinvar.

https://www.ncbi.nlm.nih.gov/research/litvar2/.

https://deafnessvariationdatabase.org/.

## Supplementary Information

Supplementary Material 1.

## Data Availability

The datasets supporting the conclusions of this article are included within the article and its additional files. The identified variants have been submitted to ClinVar under accession IDs SUB15415516 and SUB15436450.

## Abbreviations

BOS1: Branchio-Otic Syndrome 1
CSS4: Coffin-Siris syndrome 4
DEAPLE: Adult-onset progressive leukoencephalopathy with deafness
HL: Hearing loss
KBGS: KBG syndrome
KS: Kabuki syndrome 1
LEPID: Infantile-onset progressive leukoencephalopathy with or without deafness
LP: Likely pathogenic
MICPCH: Intellectual developmental disorder with microcephaly and pontine and cerebellar hypoplasia
MRD23: Intellectual developmental disorder, autosomal dominant 23 syndrome
NDD: Neurodevelopmental disorder
NSHL: Non-syndromic hearing loss
PCWH: Peripheral demyelinating neuropathy, central dysmyelination, Waardenburg syndrome, and Hirschsprung disease
P: Pathogenic
SHL: Syndromic hearing loss
USH: Usher syndrome
VUS: Variants of unknown significance
WHSUS: White-Sutton syndrome
WITKOS: Witteveen-Kolk syndrome
WS: Waardenburg syndrome
