# Supplementary material for: Clinical and genetic heterogeneity of syndromic hearing loss and its non-syndromic hearing loss mimics
Source: Mol Med. 2026 Apr 7;32:50. doi: 10.1186/s10020-026-01431-6 (PMC13055003; doi:10.1186/s10020-026-01431-6)
Supplement: Supplementary file 1 — Supplementary Material 1. [file 10020_2026_1431_MOESM1_ESM.docx]

**Table S1: The ACMG/AMP criteria used for each variant in Subgroup 1**

| **Gene** | **Variant** | **ACMG Criteria** | **Classification** | **Reference** |
| --- | --- | --- | --- | --- |
|  |  |  |  |  |
| *SIN3A* | c.3418C>T, p.(Arg1140*) | PVS1, PM2_Sup, PM6 | P | This study |
| NM_001145358.2 |  |  |  |  |
| *SOX10*  NM_006941.4 | c.698-4_698-2delinsATG, p.? | PVS1_Strong, PM2_Sup, PM6 | LP | This study |
|  | c.376_377insG, p.(Tyr126*) | PVS1, PM2_Sup, PM6 | LP | This study |
| *SETD5*  NM_001080517.3 | c.1333C>T, p.(Arg445*) | PVS1, PM2_Sup, PM6 | P | (Grozeva et al. 2014) |
|  | c.2182del, p.(Asp728Ilefs*9) | PVS1, PM2_Sup, PM6 | P | This study |
| *ANKRD11* NM_013275 | c.6836_6837del p.(Val2279Glyfs*16) | PVS1, PM2_Sup, PS4_Sup | P | (Bestetti et al. 2022; Loberti et al. 2022; Peluso et al. 2023; Wang et al. 2021) |
| *SMARCA4* | c.761G>T, p.(Gly254Val) | PM2_Sup, PP3_Sup  (AlphaMissense: 0.254, CADD: 33, REVEL: 0.594) | VUS | This study |
| NM_001387283.1 |  |  |  |  |
| *RPS6KA3* | c.1741A>G, p.(Thr581Ala) | PM2_Sup, PM6, PP3_Mod,  (AlphaMissense: 0.9939, CADD: 28.5, REVEL: 0.663), PP4 | LP | (Zhou et al. 2022) |
| NM_004586.3 |  |  |  |  |
| *PTPN11* NM_002834.3 | c.5C>T, p.(Thr2Ile) | PM2_Sup, PM6, PP3_Sup  (AlphaMissense: 0.9335, CADD: 31, REVEL: 0.213), PS4_Mod | LP | (Leach et al. 2019) |
| *BRAF* | c.2204G>A, | PM2_Sup, PP3_Sup  (AlphaMissense: 0.9661, CADD: 33, REVEL: 0.476) | VUS | This study |
| NM_004333.6 | p.(Arg735Gln) |  |  |  |
| *POGZ* | c.2569A>G, p.(Arg857Gly) | PS2, PM2_Sup, PP3  (SpliceAI Δ score: 0.83 (Acceptor loss), 0.90 (Donor loss)) | LP | This study |
| NM_015100.4 |  |  |  |  |
| *KARS1* NM_001130089.2 | c.223del, p.(Gln75Serfs*2) | PVS1, PM2_Sup PP4 | P | (Cappuccio et al. 2021) |
|  | c.1028T>C, p.(Val343Ala) | PM2_Sup, PM3,  PP3_Mod (AlphaMissense: 0.9179, CADD: 28.2, REVEL: 0.876), PP4 | LP | This study |
|  | c.379T>C, p.(Phe127Leu) | PM2_Sup, PM3_Mod,  PP3_Sup (AlphaMissense: 0.992, CADD: 27.4, REVEL: 0.570), PS4_Sup | LP | (Lin et al. 2021; Ardissone et al. 2018; AlAbdi et al. 2023) |
|  | c.889C>G, p.(Pro297Ala) | PM2_Sup, PM3_Sup, PP3_Mod  (AlphaMissense: 0.841, CADD: 29, REVEL: 0.957) | VUS | (Lin et al. 2021) |
|  | c.1124A>G, p.(Tyr375Cys) | PM2_Sup, PM3_Mod, PP3_Mod  (CADD: 31, REVEL: 0.914) | VUS | (Lin et al. 2021) |
| *HSD17B4* | c.394C>T, p.(Arg132Trp) | PM2_Sup, PM3, PP3_Sup  (CADD:26.1, REVEL: 0.874), PS4_Mod | LP | (Ferdinandusse et al. 2006) |
| NM_000414.4 |  |  |  |  |
| *TWNK* | c.874C>A, p.(Pro292Thr) | PM2_Sup, PP3_Sup (AlphaMissense: 0.1908, CADD: 21.9, REVEL: 0.729) | VUS | (Jamali et al. 2019) |
| NM_021830.3 |  |  |  |  |
| *CASK*  NM_003688.3 | c.173-2331_278+1542del | PVS1, PM2_Sup, PM6 | P | This study |
| *BBS1* | c.1642del, p.(Leu548Trpfs*31) | PVS1_Strong, PM2_Sup, PP4 | LP | (Beales et al. 2003) |
| NM_024649.4 |  |  |  |  |
| *FRAS1* | c.9607G>T, p.(Asp3203Tyr) | PM2_Sup, PP3_Mod  (AlphaMissense: 0.706, CADD: 31, REVEL: 0.920), PP4 | VUS | This study |
| NM_025074.6 |  |  |  |  |
| *GALC* | c.328+1G>T, p.? | PVS1, PM2_Sup, PM3_Sup | P | (Capalbo et al. 2019) |
| NM_000153.4 |  |  |  |  |
| *PEX1* | c.3116C>T, p.(Thr1039Ile) | PM2_Sup, PP3_Sup  (AlphaMissense: 0.8202, CADD: 26.7, REVEL: 0.879) | VUS | This study |
| NM_000466.2 |  |  |  |  |
| *CACNA1D* | c.1580G>A, p.(Arg527His) | PM2_Sup, PP3_Sup  (AlphaMissense: 0.3043, CADD: 25.8, REVEL: 0.694) | VUS | This study |
| NM_000720.3 |  |  |  |  |
| *LRP2* | c.1342-2A>T, p.? | PVS1, PM2_Sup, PP4 | P | This study |
| NM_004525.2 |  |  |  |  |
| *ELOVL4* | c.424A>G, p.(Thr142Ala) | PM2_Sup, PP3_Mod  (AlphaMissense: 0.9926, CADD: 29.7, REVEL: 0.838) | VUS | (Alabdulrazzaq et al. 2023) |
| NM_022726.3 |  |  |  |  |
| *AAAS*  NM_015665.5 | c.398_399+2del. p.(Leu134Glnfs*8) | PVS1, PM2_Sup, PM3_Sup | P | (Yildirim et al. 2023) |
| *SGSH* NM_000199.5 | c.364G>A, p.(Gly122Arg) | PS3, PM1, PM2_Sup, PP3_Sup  (AlphaMissense: 0.9781, CADD: 25.7, REVEL: 0.938) | LP | (Sidhu et al. 2014) |
| *KMT2D* NM_003482.4 | c.993dup, p.(Glu332Argfs*10) | PVS1, PM2_Sup, PM6 | P | This study |
| *FGF3* NM_005247.4 | c.166C>T, p.(Leu56Phe) | PM2_Sup, PP3_Sup  (AlphaMissense: 0.9468, CADD: 26.7, REVEL: 0.788) | VUS | (Doll et al. 2020) |

Abbreviations: Sup, supporting; Mod, moderate; P, pathogenic; LP, likely pathogenic; VUS, variant of unknown significance

**Table S2: The ACMG/AMP criteria used for each variant in Subgroup 2**

| ***Gene*** | **Variant** | **ACMG Criteria** | **Classification** | **Reference** |
| --- | --- | --- | --- | --- |
|  |  |  |  |  |
| *GATA3* | c.1059delA, p.(Arg353Serfs*3) | PVS1, PM2_Sup, PM6 | P | This study |
| NM_001002295.2 |  |  |  |  |
| *POLR1D* | c.161C>A, p.(Ser54Tyr) | PM1, PM2_Sup, PP3_Mod (AlphaMissense: 0.9562, CADD: 29.1 REVEL: 0.793) | VUS | This study |
| NM_015972.3 |  |  |  |  |
| *USH2A*  NM_206933.4 | c.11864G>A, p.(Trp3955*) | PVS1, PM2_Sup, PM3_Strong | P | (van Wijk et al. 2004) |
|  | c.653T>A, p.(Val218Glu) | PM2_Sup, PM3_Strong, PP3  (AlphaMissense: 0.8241, CADD: 25.5, REVEL: 0.858) | LP | (Besnard et al. 2014) |
|  | c.2299del, p.(Glu757Serfs*21) | PVS1, PM2_Sup, PM3_Strong | P | (Aller et al. 2010) |
|  | c.920_923dup, p.(His308Glnfs*16) | PVS1, PM2_Sup, PM3_ Strong | P | (Corton et al. 2013) |
|  | c.7595-2144A>G, p.? | PM2_Sup, PM3_Very Strong, PS3_Sup | P | (Vache et al. 2012) |
|  | Exon 14 del | PVS1, PM2_Sup, PM3_Mod | P | (Glockle et al. 2014) |
|  | Exon 44 del | PVS1_Mod, PM2_Sup, PM3_Mod, PP4 | LP | (Glockle et al. 2014) |
|  | c.848+1G>T, p.? | PVS1, PM2_Mod | LP | (Mansard et al. 2021) |
| *ADGRV1*  NM_032119.3 | c.12982G>T, p.(Glu4328*) | PVS1, PM2_Sup, PP4 | P | (Turro et al. 2020) |
|  | c.4458T>G, p.(Tyr1486*) | PVS1, PM2_Sup, PP4 | P | This study |
| *MYO7A*  NM_000260.3 | c.4361T>G, p.(Val1454Gly) | PS1, PM2_Sup, PM3_Sup | LP | This study |
|  | c.6231dup, p.(Lys2078Argfs*50) | PVS1, PM2_Sup, PM3_Sup | P | (Neuhaus et al. 2017) |
|  | c.577A>C, p.(Thr193Pro) | PM2_Sup, PM3_Sup | VUS | (Sloan-Heggen et al. 2015) |
|  | c.1190C>A, p.(Ala397Asp) | PS1, PM2_Sup, PM3_Sup,  PP3_Sup (AlphaMissense: 0.9955, CADD: 26.5, REVEL: 0.942) | LP | (Watanabe et al. 2008) |
| *CDH23*  NM_022124.5 | c.2349C>A, p.(Tyr783*) | PVS1, PM2_Sup, PM3_Sup | P | This study |
|  | c.9040del, p.(Val3014*) | PVS1, PM2_Sup, PM3_Sup | P | This study |
|  | Exons 4-6 del | PVS1, PM2_Sup, PM3_Sup | P | (Weisschuh et al. 2020; Lin et al. 2024) |
|  | c.6050-70G>A, p.? | PM2_Sup, PP3 | VUS | This Study |
| *USH1C*  NM_153676.3 | c.388-­1G>C, p.? | PVS1, PS1, PM2_Sup | P | This study |
| *SOX10*  NM_006941.3 | c.373C>T, (p.Gln125*) | PVS1, PM2_Sup, PM6 | P | (Wakabayashi et al. 2021) |
|  | c.137del, p.(Pro46Argfs*63) | PVS1, PM2_Sup | LP | This study |
|  | c.378C>G, p.(Tyr126*) | PVS1, PM2_Sup, PS4_Sup | P | (Li et al. 2021) |
|  | c.230del, p.(Ser77Thrfs*32) | PVS1, PM2_Sup, PM6 | P | This study |
| *EDN3*  NM_207034.2 | c.472C>T, p.(Arg158Cys) | PM2_Sup, PP3_Sup  (AlphaMissense: 0.8773, CADD: 25, REVEL: 0.68), PP4 | VUS | This study |
| *HSD17B4*  NM_000414.4 | c.338A>T, p.(Asp113Val) | PM2_Sup, PP3_Sup  (AlphaMissense: 0.2127, CADD: 26.6, REVEL: 0.796) | VUS | This study |
| *LARS2*  *NM_015340.4* | c.1565C>A, p.(Thr522Asn) | PS3_Sup, PM2_Sup, PM3_Strong_ PP3_Sup  (AlphaMissense: 0.829, CADD: 24.9, REVEL: 0.844) | LP | (Demain et al. 2017) |
| *OPA1*  NM_130837.2 | c.1819T>C, p.(Phe607Leu) | PM2, PM6, PP3_Mod  (AlphaMissense: 0.9994, CADD: 29.4, REVEL: 0.86) | LP | This study |
| *SLC29A3*  NM_018344.5 | c.1309G>A, p.(Gly437Arg) | PM1, PM2_Sup, PM3, PP3_Sup  (AlphaMissense: 0.9613, CADD: 26.9, REVEL: 0.69) | LP | (El-Bassyouni, Thomas, and Tosson 2020) |
|  | c.610+1G>A, p.? | PVS1, PS1, PM2_Sup | P | (Rezaie et al. 2024) |
|  | c.1087C>T, (p.Arg363Trp) | PM2_Sup, PM3_Mod, PM5, PP3_Sup  (AlphaMissense: 0.6113, CADD: 27, REVEL: 0.829) | LP | (Bloom et al. 2017) |
| *COL4A3*  NM_000091.4 | c.172G>A, p.(Gly58Ser) | PM2_Sup, PP3_Sup (AlphaMissense: 0.2845, CADD: 25.4, REVEL :0.934) | VUS | (Moriniere et al. 2014) |
|  | c.4347_4353del, p.(Arg1450Valfs*77) | PVS1, PM2_Sup, PS4_Sup, PM3_Sup | P | (Storey et al. 2013; Ding et al. 1995) |
|  |  |  |  |  |
| *COL4A3*  NM_000091.4 | c.4862C>T, p.(Thr1621Met) | PM2_Sup, PP3_Sup (CADD: 26.2, REVEL: 0.801) | VUS | This study |
| *COL4A3*  NM_000091.4 | c.3882+5G>A | PS1, PS4_Sup, PM2_Sup, PP3 | LP | (Daga et al. 2018; Zhang et al. 2012) |
| *RAF1*  NM_002880 | c.1922C>T, p.(Thr641Met) | PM2_Sup, PS4_Sup, PP3 (CADD: 25.6, REVEL: 0.713) | VUS | (Dhandapany et al. 2014) |
|  | c.912G>A, p.(Trp304*) | PVS1, PM2_Sup | LP | This study |
| *KCNQ1*  NM_000218.2 | c.514G>A, p.(Val172Met) | PS2_Sup, PM2_Sup | VUS | (Bdier et al. 2017) |
|  | c.877C>T p.(Arg293Cys) | PS2_Sup, PM2_Sup | VUS | (Bdier et al. 2017) |
|  | c.683+1G>A, p.? | PVS1, PM2_Sup | LP | (e and e 2019) |
|  | c.1265dup, p.(Phe423Valfs*40) | PVS1, PS4_Sup, PM2_Sup | P | (Akgun-Dogan et al. 2022; Abolhassani et al. 2024) |
| *BSND*  NM_057176.3 | c.64G>A, p.(Gly22Ser) | PM2_Sup, PP3_Sup (AlphaMissense: 0.868, CADD: 27.2, REVEL: 0.783) | VUS | This study |
| *SLC19A2* NM_006996.2 | c.697C>T, p.(Gln233*) | PVS1, PM1, PM2_Sup | P | (Mohsen-Pour et al. 2022) |
| *LHX3*  NM_014564.5 | c.331G>A, p.(Ala111Thr) | PM2_Sup, PP3_Sup (AlphaMissense: 0.9817, CADD: 27.9, REVEL: 0.899) | VUS | This study |
|  | c.353T>G, p.(Leu118Arg) | PM2_Sup, PP3_Mod (AlphaMissense: 0.9724, CADD: 29.6, REVEL: 0.91) | VUS | This study |
| *COL9A3*  NM_001853.3 | c.355del, p.(Leu119Serfs*10) | PVS1, PM2_Sup | P | (Rad et al. 2022) |
| *PAX3*  NM_181458.4 | c.808C>T, p.(Arg270Cys) | PS1, PS4_Mod, PM2_Sup, PP3_Mod (AlphaMissense: 0.9996, CADD:32, REVEL: 0.935) | P | This study |
| *EYA1*  NM_000503.6 | c.639+98_760del, p.(Asp214Profs*112) | PVS1, PM2_Sup, PP4 | P | This study |

Abbreviations: Sup, supporting; Mod, moderate; P, pathogenic; LP, likely pathogenic; VUS, variant of unknown significance

**Table S3: The ACMG/AMP criteria used for each variant in Subgroup 3**

| ***Gene*** | **Variant** | **ACMG Criteria** | **Classification** | **Phenotypes in the literature** | **Reference** |
| --- | --- | --- | --- | --- | --- |
|  |  |  |  |  |  |
| *SLC26A4*  NM_000441.1 | c.1001+1G>A, p.? | PVS1, PM2_Sup, PM3_Strong | P | Pendred, HL with enlarged vestibular aqueduct, NSHL | (Nakano et al. 2022) (de Moraes et al. 2013) (Mikkelsen, Tranebjaerg, and Mey 2019) |
|  | c.1334T>G, p.(Leu445Trp) | PM2_Sup, PM3_ Very Strong, PP3_Mod  (AlphaMissense: 0.9933, CADD: 29.3, REVEL: 0.965) | P | Pendred, HL with enlarged vestibular aqueduct, NSHL | (Smits et al. 2022) (Cengiz et al. 2017) (Rebeh et al. 2010; Ladsous et al. 2014) |
|  |  |  |  |  |  |
|  | c.919-2A>G, p.? | PVS1, PM2, PM3_Strong | P | Pendred, HL with enlarged vestibular aqueduct | (Zhao et al. 2024) (Nakano et al. 2022; Li et al. 2012) (Cengiz et al. 2017) (Yang et al. 2005) |
|  |  |  |  |  |  |
|  | c.2027T>A, p.(Leu676Gln) | PM2_Sup, PM3_ Very Strong, PP3_Sup  (AlphaMissense: 0.8935, CADD: 26.3, REVEL: 0.733) | P | Pendred, HL with enlarged vestibular aqueduct | (Yoon et al. 2008) (Liu et al. 2021) (Lu et al. 2022) (Baldyga et al. 2023) |
|  | c.578C>T, p.(The193Ile) | PM2_Sup, PS3_Sup, PM3_ Strong, PP3_Sup  (AlphaMissense: 0.9082, CADD: 23, REVEL: 0.854) | LP | HL with enlarged vestibular aqueduct, NSHL | (de Moraes et al. 2016) (Cengiz et al. 2017) |
|  | c.1238A>G, p.(Gln413Arg) | PS1, PM2_Sup, PS3_Sup, PM3_ Strong, PP3_Sup (AlphaMissense: 0.8859, CADD: 26.7, REVEL: 0.949) | P | Pendred, HL with enlarged vestibular aqueduct, NSHL | (de Moraes et al. 2016) (Kuhnen et al. 2014) (Huang et al. 2011) (Wang et al. 2014) |
|  | c.716T>A, p.(Val239Asp) | PM2_Sup, PM3_Very Strong, PP3_Sup  (AlphaMissense: 0.9612, CADD: 27, REVEL: 0.935) | P | Pendred, NSHL | (Walsh et al. 2006) (Soh et al. 2015) (Azadegan-Dehkordi et al. 2018) |
|  | c.170C>A, p.(Ser57*) | PVS1, PM2_Sup, PM3_Mod | P | HL with enlarged vestibular aqueduct | (Kinoglu et al. 2020) |
|  | c.212T>A, p.(Ile71Asn) | PM2_Sup, PM3_Mod, PP3_Sup  (AlphaMissense: 0.7472, CADD: 25.2, REVEL: 0.646) | VUS | Pendred | (Said et al. 2022) |
|  | c.415+2T>C, p.? | PVS1, PM2_Sup, PM3_Mod | P | HL with enlarged vestibular aqueduct | (Yuan et al. 2012) (Tian et al. 2021) |
|  | c.704A>G, p.(Gln235Arg) | PM2_Sup, PM3_ Strong, PP3_Sup  (AlphaMissense: 0.9444, CADD: 26.3, REVEL: 0.969) | LP | Pendred | (Tesolin et al. 2021) |
|  | c.1223C>A, p.(Ser408Tyr) | PM2_Sup, PP3_Mod_PM5  (AlphaMissense: 0.9937, CADD: 29.5, REVEL: 0.957) | VUS | This study | This study |
| *SLC26A4*  NM_000441.1 | c.2106del, p.(Lys702Asnfs*19) | PVS1, PM2_Sup, PM3_Sup | P | HL with enlarged vestibular aqueduct | (Yazdanpanahi et al. 2012) (Zhao et al. 2019) |
|  | c.845G>A, p.(Cys282Tyr) | PM2_Sup, PM3_ Strong,  PP3_Sup (AlphaMissense: 0.9444, CADD:26.3, REVEL: 0.969) | LP | HL with enlarged vestibular aqueduct | (Kinoglu et al. 2020) (Baldyga et al. 2023) |
|  | c.1028_1029insTCAG, p.(Ser344Glnfs*34) | PVS1, PS1, PM2_Sup | P | This study | This study |
|  | c.1001G>T, p.(Gly334Val) | PS1, PM2_Sup, PM3_ Strong, PP3_Sup (AlphaMissense: 0.7329, CADD: 35, REVEL: 0.961,  SpliceAI Δ scores: Donor Loss: 0.46, Donor Gain: 0.64) | P | HL with enlarged vestibular aqueduct | (Cengiz et al. 2017) |
|  | c.1234G>T, p.(Val412Phe) | PM2_Sup, PM3_Mod,  PP3_Mod (AlphaMissense: 0.8251, CADD:29.7,REVEL: 0.937) | LP | HL no more comments | (Azaiez et al. 2018) |
|  | c.1198del, p.(Cys400Valfs*32) | PVS1, PM2_Sup, PM3_Sup | P | HL with enlarged vestibular aqueduct | (Cengiz et al. 2017) |
| *CDH23* NM_022124.5 | c.2398-1G>T, p.? | PVS1, PM2_Sup, PM3_Sup | P | Retinal disease | (Ellingford et al. 2016) |
| *USH1C* NM_153676 | c.463C>T, p.(Arg155*) | PVS1, PM2_Sup, PM3_Sup | P | Usher syndrome | (Huang et al. 2015) (Bonnet et al. 2016) (Hanany, Rivolta, and Sharon 2020) |

Abbreviations: Sup, supporting; Mod, moderate; P, pathogenic; LP, likely pathogenic; VUS, variant of unknown significance; HL, hearing loss; NSHL, non-syndromic hearing loss

**Table S4: Sequencing methods and analysis summaries**

| **Patient ID** | **Center** | **Method** | **Data analysis and filtering strategy** |
| --- | --- | --- | --- |
| Patient 1-22, 57, 59 | Würzburg | WES | Koparir et al., 2024 (Koparir et al. 2024) |
| Patient 23-29 | Würzburg | WGS | Bengl et al., 2025 (Bengl et al. 2025) |
| Patient 32-38 | Istanbul | WES | Anitha et al., 2024 (Anitha et al. 2024) |
| Patient 39-56,58 | Tübingen/Göttingen | WES | Redfield et al., 2024 (Redfield et al. 2024) |
| Patient 60-75 | Würzburg | WES | Bahena et al., 2022 (Bahena et al. 2022) |
| Patient 76-104 | Tübingen/Göttingen | WES | Redfield et al.,2022 (Redfield et al. 2024) |
| Patient 105-108 | Tübingen | MIP | Reurink et al., 2021 (Reurink et al. 2021) |
| Patient 109,110 | Tübingen | WGS | Falb et al., 2023 (Falb et al. 2023) |
| Patient 111-114 | Tübingen/Göttingen | Sanger sequencing | Shadab et al., 2025 (Shadab et al. 2025) |

WES, whole-exome sequencing; WGS, whole-genome sequencing; MIP, Molecular inversion probe; NA, not available.

**Table S5. Molecular inversion probe 89 gene panel content**

| Gene name | Gene name | Gene name | Gene name | Gene name | Gene name |
| --- | --- | --- | --- | --- | --- |
| *ACTG1* | *DCDC2* | *GRM8* | *MYH9* | *POU3F4* | *TECTA* |
| *ADCY1* | *DIABLO* | *GRXCR1* | *MYO15A* | *POU4F3* | *TJP2* |
| *BDP1* | *DIAPH1* | *GRXCR2* | *MYO3A* | *PRPS1* | *TMC1* |
| *BSND* | *ELMOD3* | *GSDME (DFNA5)* | *MYO6* | *PTPRQ* | *TMEM132E* |
| *CABP2* | *EPS8* | *HGF* | *MYO7A* | *RDX* | *TMIE* |
| *CCDC50* | *ESPN* | *ILDR1* | *NAT2* | *RIPOR2 (FAM65B)* | *TMPRSS3* |
| *CDH23* | *ESRRB* | *KARS* | *OSBPL2* | *SERPINB6* | *TNC* |
| *CEACAM16* | *EYA4* | *KCNQ4* | *OTOA* | *SIX1* | *TPRN* |
| *CIB2* | *GIPC3* | *LHFPL5* | *OTOF* | *SLC17A8* | *TRIOBP* |
| *CLDN14* | *GJB2* | *LOXHD1* | *OTOG* | *SLC26A4* | *TSPEAR* |
| *CLIC5* | *GJB3* | *LRTOMT* | *OTOGL* | *SLC26A5* | *USH1C* |
| *COCH* | *GJB6* | *MARVELD2* | *P2RX2* | *SMPX* | *USH1G* |
| *COL11A2* | *GPSM2* | *MIR96* | *PCDH15* | *STRC* | *WHRN (DFNB31)* |
| *COL4A6* | *GRHL2* | *MSRB3* | *PJVK (DFNB59)* | *SYNE4* | *WFS1* |
| *CRYM* | *GRM7* | *MYH14* | *PNPT1* | *TBC1D24* |  |

**References**

Abolhassani, A., Z. Fattahi, M. Beheshtian, M. Fadaee, R. Vazehan, F. Ahangari, S. Dehdahsi, M. Faraji Zonooz, E. Parsimehr, Z. Kalhor, F. Peymani, M. Mozaffarpour Nouri, M. Babanejad, K. Noudehi, F. Fatehi, S. Zamanian Najafabadi, F. Afroozan, H. Yazdan, B. Bozorgmehr, A. Azarkeivan, S. Sadat Mahdavi, P. Nikuei, F. Fatehi, P. Jamali, M. R. Ashrafi, P. Karimzadeh, H. Habibi, K. Kahrizi, S. Nafissi, A. Kariminejad, and H. Najmabadi. 2024. 'Clinical application of next generation sequencing for Mendelian disease diagnosis in the Iranian population', *NPJ Genom Med*, 9: 12.

Akgun-Dogan, O., N. B. Agaoglu, K. Demirkol Y, L. Doganay, Y. Ergul, and M. Karacan. 2022. 'Mutational spectrum of congenital long QT syndrome in Turkey; identification of 12 novel mutations across KCNQ1, KCNH2, SCN5A, KCNJ2, CACNA1C, and CALM1', *J Cardiovasc Electrophysiol*, 33: 262-73.

AlAbdi, L., S. Maddirevula, H. E. Shamseldin, E. Khouj, R. Helaby, H. Hamid, A. Almulhim, M. O. Hashem, F. Abdulwahab, O. Abouyousef, M. Alqahtani, N. Altuwaijri, A. Jaafar, T. Alshidi, F. Alzahrani, Group Mendeliome, and F. S. Alkuraya. 2023. 'Diagnostic implications of pitfalls in causal variant identification based on 4577 molecularly characterized families', *Nat Commun*, 14: 5269.

Alabdulrazzaq, F., T. Alanzi, H. H. Al-Balool, A. Gardham, E. Wakeling, H. G. Leitch, M. AlSayed, M. Abdulrahim, A. Aladwani, A. Romito, K. Kampe, S. Ferdinandusse, A. H. Aboelanine, A. Abdullah, A. Alwadani, L. Bastaki, F. M. Vaz, A. M. Bertoli-Avella, and D. Marafi. 2023. 'Expanding the allelic spectrum of ELOVL4-related autosomal recessive neuro-ichthyosis', *Mol Genet Genomic Med*, 11: e2256.

Aller, E., L. Larrieu, T. Jaijo, D. Baux, C. Espinos, F. Gonzalez-Candelas, C. Najera, F. Palau, M. Claustres, A. F. Roux, and J. M. Millan. 2010. 'The USH2A c.2299delG mutation: dating its common origin in a Southern European population', *Eur J Hum Genet*, 18: 788-93.

Anitha, A., M. Banerjee, I. Thanseem, A. Prakash, N. Melempatt, P. S. Sumitha, M. Iype, and S. V. Thomas. 2024. 'Rare Pathogenic Variants Identified in Whole Exome Sequencing of Monozygotic Twins With Autism Spectrum Disorder', *Pediatr Neurol*, 158: 113-23.

Ardissone, A., D. Tonduti, A. Legati, E. Lamantea, R. Barone, I. Dorboz, O. Boespflug-Tanguy, G. Nebbia, M. Maggioni, B. Garavaglia, I. Moroni, L. Farina, A. Pichiecchio, S. Orcesi, L. Chiapparini, and D. Ghezzi. 2018. 'KARS-related diseases: progressive leukoencephalopathy with brainstem and spinal cord calcifications as new phenotype and a review of literature', *Orphanet J Rare Dis*, 13: 45.

Azadegan-Dehkordi, F., R. Ahmadi, T. Bahrami, N. Yazdanpanahi, E. Farrokhi, M. A. Tabatabaiefar, and M. Hashemzadeh-Chaleshtori. 2018. 'A novel variant of SLC26A4 and first report of the c.716T>A variant in Iranian pedigrees with non-syndromic sensorineural hearing loss', *Am J Otolaryngol*, 39: 719-25.

Azaiez, H., K. T. Booth, S. S. Ephraim, B. Crone, E. A. Black-Ziegelbein, R. J. Marini, A. E. Shearer, C. M. Sloan-Heggen, D. Kolbe, T. Casavant, M. J. Schnieders, C. Nishimura, T. Braun, and R. J. H. Smith. 2018. 'Genomic Landscape and Mutational Signatures of Deafness-Associated Genes', *Am J Hum Genet*, 103: 484-97.

Bahena, P., N. Daftarian, R. Maroofian, P. Linares, D. Villalobos, M. Mirrahimi, A. Rad, J. Doll, M. A. H. Hofrichter, A. Koparir, T. Roder, S. Han, H. Sabbaghi, H. Ahmadieh, H. Behboudi, C. Villanueva-Mendoza, V. Cortes-Gonzalez, R. Zamora-Ortiz, S. Kohl, L. Kuehlewein, H. Darvish, E. Alehabib, M. L. Arenas-Sordo, F. Suri, B. Vona, and T. Haaf. 2022. 'Unraveling the genetic complexities of combined retinal dystrophy and hearing impairment', *Hum Genet*, 141: 785-803.

Baldyga, N., D. Ozieblo, N. Gan, M. Furmanek, M. L. Leja, H. Skarzynski, and M. Oldak. 2023. 'The Genetic Background of Hearing Loss in Patients with EVA and Cochlear Malformation', *Genes (Basel)*, 14.

Bdier, A. Y., S. Al-Ghamdi, P. K. Verma, K. Dagriri, B. Alshehri, O. A. Jiman, S. E. Ahmed, A. A. M. Wilde, Z. A. Bhuiyan, and J. Y. Al-Aama. 2017. 'Autosomal recessive long QT syndrome, type 1 in eight families from Saudi Arabia', *Mol Genet Genomic Med*, 5: 592-601.

Beales, P. L., J. L. Badano, A. J. Ross, S. J. Ansley, B. E. Hoskins, B. Kirsten, C. A. Mein, P. Froguel, P. J. Scambler, R. A. Lewis, J. R. Lupski, and N. Katsanis. 2003. 'Genetic interaction of BBS1 mutations with alleles at other BBS loci can result in non-Mendelian Bardet-Biedl syndrome', *Am J Hum Genet*, 72: 1187-99.

Bengl, D., A. Koparir, W. E. Prastyo, C. Remmele, M. Dittrich, S. Flandin, W. Shehata-Dieler, C. Grimm, T. Haaf, and M. A. H. Hofrichter. 2025. 'Whole-genome sequencing, as a powerful diagnostic tool in hearing loss, reveals novel variants in PTPRQ missed by whole-exome sequencing', *BMC Med Genomics*, 18: 59.

Besnard, T., G. Garcia-Garcia, D. Baux, C. Vache, V. Faugere, L. Larrieu, S. Leonard, J. M. Millan, S. Malcolm, M. Claustres, and A. F. Roux. 2014. 'Experience of targeted Usher exome sequencing as a clinical test', *Mol Genet Genomic Med*, 2: 30-43.

Bestetti, I., M. Crippa, A. Sironi, F. Tumiatti, M. Masciadri, M. F. Smeland, S. Naik, O. Murch, M. T. Bonati, A. Spano, E. Cattaneo, M. Mariani, F. Gotta, F. Crosti, P. Cavalli, C. Pantaleoni, F. Natacci, M. F. Bedeschi, D. Milani, S. Maitz, A. Selicorni, L. Spaccini, A. Peron, S. Russo, L. Larizza, K. Low, and P. Finelli. 2022. 'Expanding the Molecular Spectrum of ANKRD11 Gene Defects in 33 Patients with a Clinical Presentation of KBG Syndrome', *Int J Mol Sci*, 23.

Bloom, J. L., C. Lin, L. Imundo, S. Guthery, S. Stepenaskie, C. Galambos, A. Lowichik, and J. F. Bohnsack. 2017. 'H syndrome: 5 new cases from the United States with novel features and responses to therapy', *Pediatr Rheumatol Online J*, 15: 76.

Bonnet, C., Z. Riahi, S. Chantot-Bastaraud, L. Smagghe, M. Letexier, C. Marcaillou, G. M. Lefevre, J. P. Hardelin, A. El-Amraoui, A. Singh-Estivalet, S. Mohand-Said, S. Kohl, A. Kurtenbach, I. Sliesoraityte, D. Zobor, S. Gherbi, F. Testa, F. Simonelli, S. Banfi, A. Fakin, D. Glavac, M. Jarc-Vidmar, A. Zupan, S. Battelino, L. Martorell Sampol, M. A. Claveria, J. Catala Mora, S. Dad, L. B. Moller, J. Rodriguez Jorge, M. Hawlina, A. Auricchio, J. A. Sahel, S. Marlin, E. Zrenner, I. Audo, and C. Petit. 2016. 'An innovative strategy for the molecular diagnosis of Usher syndrome identifies causal biallelic mutations in 93% of European patients', *Eur J Hum Genet*, 24: 1730-38.

Capalbo, A., R. A. Valero, J. Jimenez-Almazan, P. M. Pardo, M. Fabiani, D. Jimenez, C. Simon, and J. M. Rodriguez. 2019. 'Optimizing clinical exome design and parallel gene-testing for recessive genetic conditions in preconception carrier screening: Translational research genomic data from 14,125 exomes', *PLoS Genet*, 15: e1008409.

Cappuccio, G., C. Ceccatelli Berti, E. Baruffini, J. Sullivan, V. Shashi, T. Jewett, T. Stamper, S. Maitz, F. Canonico, A. Revah-Politi, G. S. Kupchik, K. Anyane-Yeboa, V. Aggarwal, A. Benneche, E. Bratland, S. Berland, F. D'Arco, C. A. Alves, A. Vanderver, D. Longo, E. Bertini, A. Torella, V. Nigro, Program Telethon Undiagnosed Diseases, A. D'Amico, M. S. van der Knaap, P. Goffrini, and N. Brunetti-Pierri. 2021. 'Bi-allelic KARS1 pathogenic variants affecting functions of cytosolic and mitochondrial isoforms are associated with a progressive and multisystem disease', *Hum Mutat*, 42: 745-61.

Cengiz, F. B., R. Yilmazer, L. Olgun, L. Sennaroglu, T. Kirazli, H. Alper, Y. Olgun, A. Incesulu, T. Atik, F. Huesca-Hernandez, J. Dominguez-Aburto, G. Gonzalez-Rosado, E. Hernandez-Zamora, M. L. Arenas-Sordo, I. Menendez, K. S. Orhan, H. Avci, N. Mahdieh, M. Bonyadi, J. Foster, 2nd, D. Duman, F. Ozkinay, S. H. Blanton, G. Bademci, and M. Tekin. 2017. 'Novel pathogenic variants underlie SLC26A4-related hearing loss in a multiethnic cohort', *Int J Pediatr Otorhinolaryngol*, 101: 167-71.

Corton, M., K. M. Nishiguchi, A. Avila-Fernandez, K. Nikopoulos, R. Riveiro-Alvarez, S. D. Tatu, C. Ayuso, and C. Rivolta. 2013. 'Exome sequencing of index patients with retinal dystrophies as a tool for molecular diagnosis', *PLoS One*, 8: e65574.

Daga, S., M. Baldassarri, C. Lo Rizzo, C. Fallerini, V. Imperatore, I. Longo, E. Frullanti, E. Landucci, L. Massella, C. Pecoraro, G. Garosi, F. Ariani, M. A. Mencarelli, F. Mari, A. Renieri, and A. M. Pinto. 2018. 'Urine-derived podocytes-lineage cells: A promising tool for precision medicine in Alport Syndrome', *Hum Mutat*, 39: 302-14.

de Moraes, V. C., N. Z. dos Santos, P. Z. Ramos, M. C. Svidnicki, A. M. Castilho, and E. L. Sartorato. 2013. 'Molecular analysis of SLC26A4 gene in patients with nonsyndromic hearing loss and EVA: identification of two novel mutations in Brazilian patients', *Int J Pediatr Otorhinolaryngol*, 77: 410-3.

de Moraes, V. C. S., E. Bernardinelli, N. Zocal, J. A. Fernandez, C. Nofziger, A. M. Castilho, E. L. Sartorato, M. Paulmichl, and S. Dossena. 2016. 'Reduction of Cellular Expression Levels Is a Common Feature of Functionally Affected Pendrin (SLC26A4) Protein Variants', *Mol Med*, 22: 41-53.

Demain, L. A., J. E. Urquhart, J. O'Sullivan, S. G. Williams, S. S. Bhaskar, E. M. Jenkinson, C. M. Lourenco, A. Heiberg, S. H. Pearce, S. A. Shalev, W. W. Yue, S. Mackinnon, K. J. Munro, R. Newbury-Ecob, K. Becker, M. J. Kim, O' Keefe RT, and W. G. Newman. 2017. 'Expanding the genotypic spectrum of Perrault syndrome', *Clin Genet*, 91: 302-12.

Dhandapany, P. S., M. A. Razzaque, U. Muthusami, S. Kunnoth, J. J. Edwards, S. Mulero-Navarro, I. Riess, S. Pardo, J. Sheng, D. S. Rani, B. Rani, P. Govindaraj, E. Flex, T. Yokota, M. Furutani, T. Nishizawa, T. Nakanishi, J. Robbins, G. Limongelli, R. J. Hajjar, D. Lebeche, A. Bahl, M. Khullar, A. Rathinavel, K. C. Sadler, M. Tartaglia, R. Matsuoka, K. Thangaraj, and B. D. Gelb. 2014. 'RAF1 mutations in childhood-onset dilated cardiomyopathy', *Nat Genet*, 46: 635-39.

Ding, J., J. Stitzel, P. Berry, E. Hawkins, and C. E. Kashtan. 1995. 'Autosomal recessive Alport syndrome: mutation in the COL4A3 gene in a woman with Alport syndrome and posttransplant antiglomerular basement membrane nephritis', *J Am Soc Nephrol*, 5: 1714-7.

Doll, J., B. Vona, L. Schnapp, F. Ruschendorf, I. Khan, S. Khan, N. Muhammad, S. Alam Khan, H. Nawaz, A. Khan, N. Ahmad, S. M. Kolb, L. Kuhlewein, J. D. J. Labonne, L. C. Layman, M. A. H. Hofrichter, T. Roder, M. Dittrich, T. Muller, T. D. Graves, I. K. Kong, I. Nanda, H. G. Kim, and T. Haaf. 2020. 'Genetic Spectrum of Syndromic and Non-Syndromic Hearing Loss in Pakistani Families', *Genes (Basel)*, 11.

e, Merge Consortium Electronic address agibbs bcm edu, and Merge Consortium e. 2019. 'Harmonizing Clinical Sequencing and Interpretation for the eMERGE III Network', *Am J Hum Genet*, 105: 588-605.

El-Bassyouni, H. T., M. M. Thomas, and A. M. S. Tosson. 2020. 'Mutation in the SLC29A3 Gene in an Egyptian Patient with H Syndrome: A Case Report and Review of Literature', *J Pediatr Genet*, 9: 109-13.

Ellingford, J. M., S. Barton, S. Bhaskar, J. O'Sullivan, S. G. Williams, J. A. Lamb, B. Panda, P. I. Sergouniotis, R. L. Gillespie, S. P. Daiger, G. Hall, T. Gale, I. C. Lloyd, P. N. Bishop, S. C. Ramsden, and G. C. M. Black. 2016. 'Molecular findings from 537 individuals with inherited retinal disease', *J Med Genet*, 53: 761-67.

Falb, R. J., A. J. Muller, W. Klein, M. Grimmel, U. Grasshoff, S. Spranger, P. Stobe, D. Gauck, A. Kuechler, N. Dikow, E. M. C. Schwaibold, C. Schmidt, L. Averdunk, R. Buchert, T. Heinrich, N. Prodan, J. Park, M. Kehrer, M. Sturm, O. Kelemen, S. Hartmann, D. Horn, D. Emmerich, N. Hirt, A. Neumann, G. Kristiansen, U. Gembruch, S. Haen, R. Siebert, S. Hentze, M. Hoopmann, S. Ossowski, S. Waldmuller, S. Beck-Wodl, D. Glaser, I. Tekesin, F. Distelmaier, O. Riess, K. O. Kagan, A. Dufke, and T. B. Haack. 2023. 'Bi-allelic loss-of-function variants in KIF21A cause severe fetal akinesia with arthrogryposis multiplex', *J Med Genet*, 60: 48-56.

Ferdinandusse, S., M. S. Ylianttila, J. Gloerich, M. K. Koski, W. Oostheim, H. R. Waterham, J. K. Hiltunen, R. J. Wanders, and T. Glumoff. 2006. 'Mutational spectrum of D-bifunctional protein deficiency and structure-based genotype-phenotype analysis', *Am J Hum Genet*, 78: 112-24.

Glockle, N., S. Kohl, J. Mohr, T. Scheurenbrand, A. Sprecher, N. Weisschuh, A. Bernd, G. Rudolph, M. Schubach, C. Poloschek, E. Zrenner, S. Biskup, W. Berger, B. Wissinger, and J. Neidhardt. 2014. 'Panel-based next generation sequencing as a reliable and efficient technique to detect mutations in unselected patients with retinal dystrophies', *Eur J Hum Genet*, 22: 99-104.

Grozeva, D., K. Carss, O. Spasic-Boskovic, M. J. Parker, H. Archer, H. V. Firth, S. M. Park, N. Canham, S. E. Holder, M. Wilson, A. Hackett, M. Field, J. A. Floyd, Uk K. Consortium, M. Hurles, and F. L. Raymond. 2014. 'De novo loss-of-function mutations in SETD5, encoding a methyltransferase in a 3p25 microdeletion syndrome critical region, cause intellectual disability', *Am J Hum Genet*, 94: 618-24.

Hanany, M., C. Rivolta, and D. Sharon. 2020. 'Worldwide carrier frequency and genetic prevalence of autosomal recessive inherited retinal diseases', *Proc Natl Acad Sci U S A*, 117: 2710-16.

Huang, S., D. Han, Y. Yuan, G. Wang, D. Kang, X. Zhang, X. Yan, X. Meng, M. Dong, and P. Dai. 2011. 'Extremely discrepant mutation spectrum of SLC26A4 between Chinese patients with isolated Mondini deformity and enlarged vestibular aqueduct', *J Transl Med*, 9: 167.

Huang, X. F., F. Huang, K. C. Wu, J. Wu, J. Chen, C. P. Pang, F. Lu, J. Qu, and Z. B. Jin. 2015. 'Genotype-phenotype correlation and mutation spectrum in a large cohort of patients with inherited retinal dystrophy revealed by next-generation sequencing', *Genet Med*, 17: 271-8.

Jamali, F., H. Ghaedi, A. Tafakhori, E. Alehabib, M. Chapi, N. Daftarian, H. Darvish, and J. Jamshidi. 2019. 'Homozygous Mutation in TWNK Cases Ataxia, Sensorineural Hearing Loss and Optic Nerve Atrophy', *Arch Iran Med*, 22: 728-30.

Kinoglu, K., K. S. Orhan, H. Kara, O. Ozturk, B. Polat, H. Aydogan, M. Celik, A. B. Ceviz, and Y. Guldiken. 2020. 'Investigation of DFNB4 SLC26A4 mutation in patients with enlarged vestibular aquaduct', *Int J Pediatr Otorhinolaryngol*, 138: 110379.

Koparir, A., C. Lekszas, K. Keseroglu, T. Rose, L. Rappl, A. Rad, R. Maroofian, N. Narendran, A. Hasanzadeh, E. G. Karimiani, F. Boschann, U. Kornak, E. Klopocki, E. M. Ozbudak, B. Vona, T. Haaf, and D. Liedtke. 2024. 'Zebrafish as a model to investigate a biallelic gain-of-function variant in MSGN1, associated with a novel skeletal dysplasia syndrome', *Hum Genomics*, 18: 23.

Kuhnen, P., S. Turan, S. Frohler, T. Guran, S. Abali, H. Biebermann, A. Bereket, A. Gruters, W. Chen, and H. Krude. 2014. 'Identification of PENDRIN (SLC26A4) mutations in patients with congenital hypothyroidism and "apparent" thyroid dysgenesis', *J Clin Endocrinol Metab*, 99: E169-76.

Ladsous, M., V. Vlaeminck-Guillem, V. Dumur, C. Vincent, F. Dubrulle, C. M. Dhaenens, and J. L. Wemeau. 2014. 'Analysis of the thyroid phenotype in 42 patients with Pendred syndrome and nonsyndromic enlargement of the vestibular aqueduct', *Thyroid*, 24: 639-48.

Leach, N. T., D. R. Wilson Mathews, L. S. Rosenblum, Z. Zhou, H. Zhu, and R. A. Heim. 2019. 'Comparative assessment of gene-specific variant distribution in prenatal and postnatal cohorts tested for Noonan syndrome and related conditions', *Genet Med*, 21: 417-25.

Li, Q., Q. W. Zhu, Y. Y. Yuan, S. S. Huang, D. Y. Han, D. L. Huang, and P. Dai. 2012. 'Identification of SLC26A4 c.919-2A>G compound heterozygosity in hearing-impaired patients to improve genetic counseling', *J Transl Med*, 10: 225.

Li, Y., J. Su, J. Zhang, J. Pei, D. Li, Y. Zhang, J. Li, M. Chen, and B. Zhu. 2021. 'Targeted next-generation sequencing of deaf patients from Southwestern China', *Mol Genet Genomic Med*, 9: e1660.

Lin, S. J., B. Vona, P. G. Barbalho, R. Kaiyrzhanov, R. Maroofian, C. Petree, M. Severino, V. Stanley, P. Varshney, P. Bahena, F. Alzahrani, A. Alhashem, A. T. Pagnamenta, G. Aubertin, J. I. Estrada-Veras, H. A. D. Hernandez, N. Mazaheri, A. Oza, J. Thies, D. L. Renaud, S. Dugad, J. McEvoy, T. Sultan, L. S. Pais, B. Tabarki, D. Villalobos-Ramirez, A. Rad, Consortium Genomics England Research, H. Galehdari, F. Ashrafzadeh, A. Sahebzamani, K. Saeidi, E. Torti, H. Z. Elloumi, S. Mora, T. B. Palculict, H. Yang, J. D. Wren, Fowler Ben, M. Joshi, M. Behra, S. M. Burgess, S. K. Nath, M. G. Hanna, M. Kenna, J. L. Merritt, 2nd, H. Houlden, E. G. Karimiani, M. S. Zaki, T. Haaf, F. S. Alkuraya, J. G. Gleeson, and G. K. Varshney. 2021. 'Biallelic variants in KARS1 are associated with neurodevelopmental disorders and hearing loss recapitulated by the knockout zebrafish', *Genet Med*, 23: 1933-43.

Lin, S., S. Vermeirsch, N. Pontikos, M. P. Martin-Gutierrez, M. Daich Varela, S. Malka, E. Schiff, H. Knight, G. Wright, N. Jurkute, M. J. Simcoe, P. Yu-Wai-Man, M. Moosajee, M. Michaelides, O. A. Mahroo, A. R. Webster, and G. Arno. 2024. 'Spectrum of Genetic Variants in the Most Common Genes Causing Inherited Retinal Disease in a Large Molecularly Characterized United Kingdom Cohort', *Ophthalmol Retina*, 8: 699-709.

Liu, Y., Z. Huang, C. Sun, X. Shen, W. Li, and Q. Li. 2021. '[Difference of SLC26A4 gene mutation frequency between patients with large vestibular aqueduct syndrome and/or Mondini dysplasia]', *Lin Chuang Er Bi Yan Hou Tou Jing Wai Ke Za Zhi*, 35: 891-95.

Loberti, L., L. P. Bruno, S. Granata, G. Doddato, S. Resciniti, F. Fava, M. Carullo, E. Rahikkala, G. Jouret, L. A. Menke, D. Lederer, P. Vrielynck, L. Ryba, N. Brunetti-Pierri, A. Lasa-Aranzasti, A. M. Cueto-Gonzalez, L. Trujillano, I. Valenzuela, E. F. Tizzano, A. M. Spinelli, I. Bruno, A. Curro, F. Stanzial, F. Benedicenti, D. Lopergolo, F. M. Santorelli, C. Aristidou, G. A. Tanteles, I. Maystadt, T. Tkemaladze, T. Reimand, H. Lokke, K. Ounap, M. K. Haanpaa, A. Holubova, V. Zoubkova, M. Schwarz, R. Zordania, K. Muru, L. Roht, A. Tihverainen, R. Teek, U. Thomson, I. Atallah, A. Superti-Furga, S. Buoni, R. Canitano, V. Scandurra, A. Rossetti, S. Grosso, R. Battini, M. Baldassarri, M. A. Mencarelli, C. L. Rizzo, M. Bruttini, F. Mari, F. Ariani, A. Renieri, and A. M. Pinto. 2022. 'Natural history of KBG syndrome in a large European cohort', *Hum Mol Genet*, 31: 4131-42.

Lu, Y. T., L. Wang, L. L. Hou, P. P. Zheng, Q. Xu, and D. T. Deng. 2022. 'SLC26A4 mutation in Pendred syndrome with hypokalemia: A case report', *Medicine (Baltimore)*, 101: e30253.

Mansard, L., D. Baux, C. Vache, C. Blanchet, I. Meunier, M. Willems, V. Faugere, C. Baudoin, M. Moclyn, J. Bianchi, H. Dollfus, B. Gilbert-Dussardier, D. Dupin-Deguine, D. Bonneau, I. Drumare, S. Odent, X. Zanlonghi, M. Claustres, M. Koenig, V. Kalatzis, and A. F. Roux. 2021. 'The Study of a 231 French Patient Cohort Significantly Extends the Mutational Spectrum of the Two Major Usher Genes MYO7A and USH2A', *Int J Mol Sci*, 22.

Mikkelsen, K. S., L. Tranebjaerg, and K. Mey. 2019. 'Cochlear implantation in a 10-year old boy with Pendred syndrome and extremely enlarged endolymphatic sacs', *Cochlear Implants Int*, 20: 100-03.

Mohsen-Pour, N., N. Naderi, S. Ghasemi, M. Hesami, M. Maleki, and S. Kalayinia. 2022. 'Whole-Exome Sequencing Revealed a Pathogenic Nonsense Variant in the SLC19A2 Gene in an Iranian Family with Thiamine-Responsive Megaloblastic Anemia', *Lab Med*, 53: 640-50.

Moriniere, V., K. Dahan, P. Hilbert, M. Lison, S. Lebbah, A. Topa, C. Bole-Feysot, S. Pruvost, P. Nitschke, E. Plaisier, B. Knebelmann, M. A. Macher, L. H. Noel, M. C. Gubler, C. Antignac, and L. Heidet. 2014. 'Improving mutation screening in familial hematuric nephropathies through next generation sequencing', *J Am Soc Nephrol*, 25: 2740-51.

Nakano, A., Y. Arimoto, H. Mutai, K. Nara, S. Inoue, and T. Matsunaga. 2022. 'Clinical and genetic analysis of children with hearing loss and bilateral enlarged vestibular aqueducts', *Int J Pediatr Otorhinolaryngol*, 152: 110975.

Neuhaus, C., T. Eisenberger, C. Decker, S. Nagl, C. Blank, M. Pfister, I. Kennerknecht, C. Muller-Hofstede, P. Charbel Issa, R. Heller, B. Beck, K. Ruther, D. Mitter, K. Rohrschneider, U. Steinhauer, H. M. Korbmacher, D. Huhle, S. M. Elsayed, H. M. Taha, S. M. Baig, H. Stohr, M. Preising, S. Markus, F. Moeller, B. Lorenz, K. Nagel-Wolfrum, A. O. Khan, and H. J. Bolz. 2017. 'Next-generation sequencing reveals the mutational landscape of clinically diagnosed Usher syndrome: copy number variations, phenocopies, a predominant target for translational read-through, and PEX26 mutated in Heimler syndrome', *Mol Genet Genomic Med*, 5: 531-52.

Peluso, F., S. G. Caraffi, G. Contro, L. Valeri, M. Napoli, G. Carboni, A. Seth, R. Zuntini, E. Coccia, G. Astrea, A. M. Bisgaard, I. Ivanovski, S. Maitz, E. Brischoux-Boucher, M. T. Carter, M. L. Dentici, K. Devriendt, M. Bellini, M. C. Digilio, A. Doja, D. A. Dyment, S. Farholt, C. R. Ferreira, L. A. Wolfe, W. A. Gahl, M. Gnazzo, H. Goel, S. W. Gronborg, T. Hammer, L. Iughetti, T. Kleefstra, D. A. Koolen, F. R. Lepri, G. Lemire, P. Louro, G. McCullagh, S. F. Madeo, A. Milone, R. Milone, J. E. K. Nielsen, A. Novelli, C. W. Ockeloen, R. Pascarella, T. Pippucci, I. Ricca, S. P. Robertson, S. Sawyer, M. Falkenberg Smeland, S. Stegmann, C. T. Stumpel, A. Goel, J. M. Taylor, D. Barbuti, A. Soresina, M. F. Bedeschi, R. Battini, A. Cavalli, C. Fusco, M. Iascone, L. Van Maldergem, S. Venkateswaran, O. Zuffardi, S. Vergano, L. Garavelli, and A. Bayat. 2023. 'Deep phenotyping of the neuroimaging and skeletal features in KBG syndrome: a study of 53 patients and review of the literature', *J Med Genet*, 60: 1224-34.

Rad, A., M. Najafi, F. Suri, S. Abedini, S. Loum, E. G. Karimiani, N. Daftarian, D. Murphy, M. Doosti, A. Moghaddasi, H. Ahmadieh, H. Sabbaghi, M. Rajati, N. Hashemi, B. Vona, and M. Schmidts. 2022. 'Identification of three novel homozygous variants in COL9A3 causing autosomal recessive Stickler syndrome', *Orphanet J Rare Dis*, 17: 97.

Rebeh, I. B., N. Yoshimi, H. Hadj-Kacem, S. Yanohco, B. Hammami, M. Mnif, M. Araki, A. Ghorbel, H. Ayadi, S. Masmoudi, and H. Miyazaki. 2010. 'Two missense mutations in SLC26A4 gene: a molecular and functional study', *Clin Genet*, 78: 74-80.

Redfield, S. E., P. De-la-Torre, M. Zamani, H. Wang, H. Khan, T. Morris, G. Shariati, M. Karimi, M. A. Kenna, G. H. Seo, H. Xu, W. Lu, S. Naz, H. Galehdari, A. A. Indzhykulian, A. E. Shearer, and B. Vona. 2024. 'PKHD1L1, a gene involved in the stereocilia coat, causes autosomal recessive nonsyndromic hearing loss', *Hum Genet*, 143: 311-29.

Reurink, J., A. Dockery, D. Ozieblo, G. J. Farrar, M. Oldak, J. B. Ten Brink, A. A. Bergen, T. Rinne, H. G. Yntema, R. J. E. Pennings, L. I. van den Born, M. Aben, J. Oostrik, H. Venselaar, A. S. Plomp, M. I. Khan, E. van Wijk, F. P. M. Cremers, S. Roosing, and H. Kremer. 2021. 'Molecular Inversion Probe-Based Sequencing of USH2A Exons and Splice Sites as a Cost-Effective Screening Tool in USH2 and arRP Cases', *Int J Mol Sci*, 22.

Rezaie, N., N. Mansour Samaei, A. Ghorbani, N. Gholipour, S. Vosough, M. Rafigh, and A. Amini. 2024. 'A novel start-loss mutation of the SLC29A3 gene in a consanguineous family with H syndrome: clinical characteristics, in silico analysis and literature review', *BMC Med Genomics*, 17: 178.

Said, M. B., I. B. Ayed, I. Elloumi, M. Hasnaoui, A. Souissi, N. Idriss, H. Aloulou, I. Chabchoub, B. Maalej, D. Driss, and S. Masmoudi. 2022. 'Custom Next-Generation Sequencing Identifies Novel Mutations Expanding the Molecular and clinical spectrum of isolated Hearing Impairment or along with defects of the retina, the thyroid, and the kidneys', *Mol Genet Genomic Med*, 10: e1868.

Shadab, M., A. Ben-Mahmoud, L. N. Martinez Volter, A. A. Abbasi, B. Ku, A. Ejaz, Z. Latif, V. Gupta, D. Owrang, M. H. Jang, Z. Zhang, R. Mohammad, H. Houlden, H. G. Kim, and B. Vona. 2025. 'Recurrent and Novel Pathogenic Variants in Genes Involved with Hearing Loss in the Pakistani Population', *Mol Diagn Ther*, 29: 519-37.

Sidhu, N. S., K. Schreiber, K. Propper, S. Becker, I. Uson, G. M. Sheldrick, J. Gartner, R. Kratzner, and R. Steinfeld. 2014. 'Structure of sulfamidase provides insight into the molecular pathology of mucopolysaccharidosis IIIA', *Acta Crystallogr D Biol Crystallogr*, 70: 1321-35.

Sloan-Heggen, C. M., M. Babanejad, M. Beheshtian, A. C. Simpson, K. T. Booth, F. Ardalani, K. L. Frees, M. Mohseni, R. Mozafari, Z. Mehrjoo, L. Jamali, S. Vaziri, T. Akhtarkhavari, N. Bazazzadegan, N. Nikzat, S. Arzhangi, F. Sabbagh, H. Otukesh, S. M. Seifati, H. Khodaei, M. Taghdiri, N. C. Meyer, A. Daneshi, M. Farhadi, K. Kahrizi, R. J. Smith, H. Azaiez, and H. Najmabadi. 2015. 'Characterising the spectrum of autosomal recessive hereditary hearing loss in Iran', *J Med Genet*, 52: 823-9.

Smits, J. J., S. E. de Bruijn, C. P. Lanting, J. Oostrik, L. O'Gorman, T. Mantere, Doofnl Consortium, F. P. M. Cremers, S. Roosing, H. G. Yntema, E. de Vrieze, R. Derks, A. Hoischen, S. A. H. Pegge, K. Neveling, R. J. E. Pennings, and H. Kremer. 2022. 'Exploring the missing heritability in subjects with hearing loss, enlarged vestibular aqueducts, and a single or no pathogenic SLC26A4 variant', *Hum Genet*, 141: 465-84.

Soh, L. M., M. Druce, A. B. Grossman, A. M. Differ, L. Rajput, M. Bitner-Glindzicz, and M. Korbonits. 2015. 'Evaluation of genotype-phenotype relationships in patients referred for endocrine assessment in suspected Pendred syndrome', *Eur J Endocrinol*, 172: 217-26.

Storey, H., J. Savige, V. Sivakumar, S. Abbs, and F. A. Flinter. 2013. 'COL4A3/COL4A4 mutations and features in individuals with autosomal recessive Alport syndrome', *J Am Soc Nephrol*, 24: 1945-54.

Tesolin, P., S. Fiorino, S. Lenarduzzi, E. Rubinato, E. Cattaruzzi, L. Ammar, V. Castro, E. Orzan, C. Granata, D. Dell'Orco, A. Morgan, and G. Girotto. 2021. 'Pendred Syndrome, or Not Pendred Syndrome? That Is the Question', *Genes (Basel)*, 12.

Tian, Y., H. Xu, D. Liu, J. Zhang, Z. Yang, S. Zhang, H. Liu, R. Li, Y. Tian, B. Zeng, T. Li, Q. Lin, H. Wang, X. Li, W. Lu, Y. Shi, Y. Zhang, H. Zhang, C. Jiang, Y. Xu, B. Chen, J. Liu, and W. Tang. 2021. 'Increased diagnosis of enlarged vestibular aqueduct by multiplex PCR enrichment and next-generation sequencing of the SLC26A4 gene', *Mol Genet Genomic Med*, 9: e1734.

Turro, E., W. J. Astle, K. Megy, S. Graf, D. Greene, O. Shamardina, H. L. Allen, A. Sanchis-Juan, M. Frontini, C. Thys, J. Stephens, R. Mapeta, O. S. Burren, K. Downes, M. Haimel, S. Tuna, S. V. V. Deevi, T. J. Aitman, D. L. Bennett, P. Calleja, K. Carss, M. J. Caulfield, P. F. Chinnery, P. H. Dixon, D. P. Gale, R. James, A. Koziell, M. A. Laffan, A. P. Levine, E. R. Maher, H. S. Markus, J. Morales, N. W. Morrell, A. D. Mumford, E. Ormondroyd, S. Rankin, A. Rendon, S. Richardson, I. Roberts, N. B. A. Roy, M. A. Saleem, K. G. C. Smith, H. Stark, R. Y. Y. Tan, A. C. Themistocleous, A. J. Thrasher, H. Watkins, A. R. Webster, M. R. Wilkins, C. Williamson, J. Whitworth, S. Humphray, D. R. Bentley, Genomes Project Nihr BioResource for the, N. Kingston, N. Walker, J. R. Bradley, S. Ashford, C. J. Penkett, K. Freson, K. E. Stirrups, F. L. Raymond, and W. H. Ouwehand. 2020. 'Whole-genome sequencing of patients with rare diseases in a national health system', *Nature*, 583: 96-102.

Vache, C., T. Besnard, P. le Berre, G. Garcia-Garcia, D. Baux, L. Larrieu, C. Abadie, C. Blanchet, H. J. Bolz, J. Millan, C. Hamel, S. Malcolm, M. Claustres, and A. F. Roux. 2012. 'Usher syndrome type 2 caused by activation of an USH2A pseudoexon: implications for diagnosis and therapy', *Hum Mutat*, 33: 104-8.

van Wijk, E., R. J. Pennings, H. te Brinke, A. Claassen, H. G. Yntema, L. H. Hoefsloot, F. P. Cremers, C. W. Cremers, and H. Kremer. 2004. 'Identification of 51 novel exons of the Usher syndrome type 2A (USH2A) gene that encode multiple conserved functional domains and that are mutated in patients with Usher syndrome type II', *Am J Hum Genet*, 74: 738-44.

Wakabayashi, T., A. Takei, N. Okada, M. Shinohara, M. Takahashi, S. Nagashima, K. Okada, K. Ebihara, and S. Ishibashi. 2021. 'A novel SOX10 nonsense mutation in a patient with Kallmann syndrome and Waardenburg syndrome', *Endocrinol Diabetes Metab Case Rep*, 2021.

Walsh, T., A. Abu Rayan, J. Abu Sa'ed, H. Shahin, J. Shepshelovich, M. K. Lee, K. Hirschberg, M. Tekin, W. Salhab, K. B. Avraham, M. C. King, and M. Kanaan. 2006. 'Genomic analysis of a heterogeneous Mendelian phenotype: multiple novel alleles for inherited hearing loss in the Palestinian population', *Hum Genomics*, 2: 203-11.

Wang, S., H. Wei, D. Fu, X. Liu, L. Shen, S. Wu, and Y. Chen. 2021. 'Clinical and genetic characteristics of Keishi-Bukuryo-Gan syndrome: an analysis of 5 cases', *Zhejiang Da Xue Xue Bao Yi Xue Ban*, 50: 494-99.

Wang, Z. T., Y. Chen, D. Y. Chen, Y. C. Chai, X. H. Pang, L. H. Sun, X. W. Wang, T. Yang, and H. Wu. 2014. 'Mutation analysis of seven consanguineous Uyghur families with non-syndromic deafness', *Int J Pediatr Otorhinolaryngol*, 78: 1513-6.

Watanabe, S., N. Umeki, R. Ikebe, and M. Ikebe. 2008. 'Impacts of Usher syndrome type IB mutations on human myosin VIIa motor function', *Biochemistry*, 47: 9505-13.

Weisschuh, N., C. D. Obermaier, F. Battke, A. Bernd, L. Kuehlewein, F. Nasser, D. Zobor, E. Zrenner, E. Weber, B. Wissinger, S. Biskup, K. Stingl, and S. Kohl. 2020. 'Genetic architecture of inherited retinal degeneration in Germany: A large cohort study from a single diagnostic center over a 9-year period', *Hum Mutat*, 41: 1514-27.

Yang, J. J., C. C. Tsai, H. M. Hsu, J. Y. Shiao, C. C. Su, and S. Y. Li. 2005. 'Hearing loss associated with enlarged vestibular aqueduct and Mondini dysplasia is caused by splice-site mutation in the PDS gene', *Hear Res*, 199: 22-30.

Yazdanpanahi, N., M. H. Chaleshtori, M. A. Tabatabaiefar, Z. Noormohammadi, E. Farrokhi, H. Najmabadi, S. Shahbazi, and A. Hosseinipour. 2012. 'Two novel SLC26A4 mutations in Iranian families with autosomal recessive hearing loss', *Int J Pediatr Otorhinolaryngol*, 76: 845-50.

Yildirim, R., E. Unal, A. Tekmenuray-Unal, F. F. Tas, S. Ozalkak, A. Cayir, and M. N. Ozbek. 2023. 'The clinical and laboratory features of patients with triple A syndrome: a single-center experience in Turkey', *Endocrine*, 79: 376-83.

Yoon, J. S., H. J. Park, S. Y. Yoo, W. Namkung, M. J. Jo, S. K. Koo, H. Y. Park, W. S. Lee, K. H. Kim, and M. G. Lee. 2008. 'Heterogeneity in the processing defect of SLC26A4 mutants', *J Med Genet*, 45: 411-9.

Yuan, Y., W. Guo, J. Tang, G. Zhang, G. Wang, M. Han, X. Zhang, S. Yang, D. Z. He, and P. Dai. 2012. 'Molecular epidemiology and functional assessment of novel allelic variants of SLC26A4 in non-syndromic hearing loss patients with enlarged vestibular aqueduct in China', *PLoS One*, 7: e49984.

Zhang, Y., F. Wang, J. Ding, H. Zhang, D. Zhao, L. Yu, H. Xiao, Y. Yao, X. Zhong, and S. Wang. 2012. 'Genotype-phenotype correlations in 17 Chinese patients with autosomal recessive Alport syndrome', *Am J Med Genet A*, 158A: 2188-93.

Zhao, X., L. Huang, X. Wang, X. Wang, L. Zhao, X. Cheng, and Y. Ruan. 2019. 'Genotyping and audiological characteristics of infants with a single-allele SLC26A4 mutation', *Int J Pediatr Otorhinolaryngol*, 116: 153-58.

Zhao, Y., Y. Long, T. Shi, X. Ma, C. Lian, H. Wang, H. Xu, L. Yu, and X. Zhao. 2024. 'Validating the splicing effect of rare variants in the SLC26A4 gene using minigene assay', *BMC Med Genomics*, 17: 233.

Zhou, X., P. Feliciano, C. Shu, T. Wang, I. Astrovskaya, J. B. Hall, J. U. Obiajulu, J. R. Wright, S. C. Murali, S. X. Xu, L. Brueggeman, T. R. Thomas, O. Marchenko, C. Fleisch, S. D. Barns, L. G. Snyder, B. Han, T. S. Chang, T. N. Turner, W. T. Harvey, A. Nishida, B. J. O'Roak, D. H. Geschwind, Spark Consortium, J. J. Michaelson, N. Volfovsky, E. E. Eichler, Y. Shen, and W. K. Chung. 2022. 'Integrating de novo and inherited variants in 42,607 autism cases identifies mutations in new moderate-risk genes', *Nat Genet*, 54: 1305-19.
